# New Donor-Acceptor Stenhouse Adducts as Visible and Near Infrared Light Polymerization Photoinitiators

**DOI:** 10.3390/molecules25102317

**Published:** 2020-05-15

**Authors:** Guillaume Noirbent, Yangyang Xu, Aude-Héloise Bonardi, Sylvain Duval, Didier Gigmes, Jacques Lalevée, Frédéric Dumur

**Affiliations:** 1 Aix Marseille Univ, CNRS, ICR UMR 7273, F-13397 Marseille, France; guillaume.noirbent@univ-amu.fr (G.N.); didier.gigmes@univ-amu.fr (D.G.); 2 Université de Haute-Alsace, CNRS, IS2M UMR 7361, F-68100 Mulhouse, France; ahutxyy@163.com (Y.X.); aude-heloise.bonardi@uha.fr (A.-H.B.); 3 Université de Strasbourg, F-67000 Strasbourg, France; 4 Université de Lille, CNRS, Centrale Lille, Univ. Artois, UMR 8181—UCCS—Unité de Catalyse et Chimie du Solide, F-59000 Lille, France; sylvain.duval@univ-lilles.fr

**Keywords:** photoinitiators, photopolymerization, visible light, DASA, Stenhouse adducts, push-pull, free radical polymerization, cationic polymerization

## Abstract

Polymerization photoinitiators that can be activated under low light intensity and in the visible range are being pursued by both the academic and industrial communities. To efficiently harvest light and initiate a polymerization process, dyes with high molar extinction coefficients in the visible range are ideal candidates. In this field, Donor-acceptor Stenhouse Adducts (DASA) which belong to a class of recently discovered organic photochromic molecules still lack practical applications. In this work, a series of DASA-based dyes are proposed as photoinitiators for the free radical polymerization of (meth)acrylates upon exposure to a near infrared light (laser diode at 785 nm).

## 1. Introduction

During the past decades, push-pull dyes composed of an electron donor connected to an electron acceptor by means of a conjugated or a non-conjugated spacer have become a thriving research area, impelled by their potential applications ranging from non-linear optical (NLO) applications [1], organic photovoltaics (OPVs) [2], organic field effect transistors (OFETs) [3], photopolymerization [4,5,6,7,8,9,10,11] or photocatalytic degradation of dyes [12]. As interesting features, the optical properties of the dyes can be finely tuned not only by adjusting the strength of the electron-withdrawing and releasing abilities of both the donors and the acceptors used to create the chromophores but also the length of the spacer used to connect the two partners [13]. By elongating the π-conjugated spacer introduced between the electron donors and acceptors, the molar extinction coefficients of the dyes can be significantly increased while jointly inducing a redshift of the absorption spectrum. Specifically, when exposed to light irradiation and irrespective of the wavelength used to excite the molecules, no color change occurs for this first class of dyes, no isomerization or structural rearrangement occurring upon photoexcitation. Besides, in some cases, a photodegradation of the molecule can nonetheless be observed, resulting from a low photochemical stability of the dye. Conversely, for a second class of dyes named photochroms or photoswitches, a totally different behavior is observed. Notably, the dyes can undergo a complete discoloration upon photoexcitation with the appropriate wavelength [14]. As specificity, the color change is reversible and can be triggered by exciting the molecules at a second irradiation wavelength carefully selected. In this field, the most popular photoswitches include azobenzenes [15], diarylethenes [16], dihydroazulenes [17] or spiropyrans [18]. For these dyes, the stable form is a colorless state and use of a high energy (UV) light is required to produce the colored form.

**Scheme 1:**
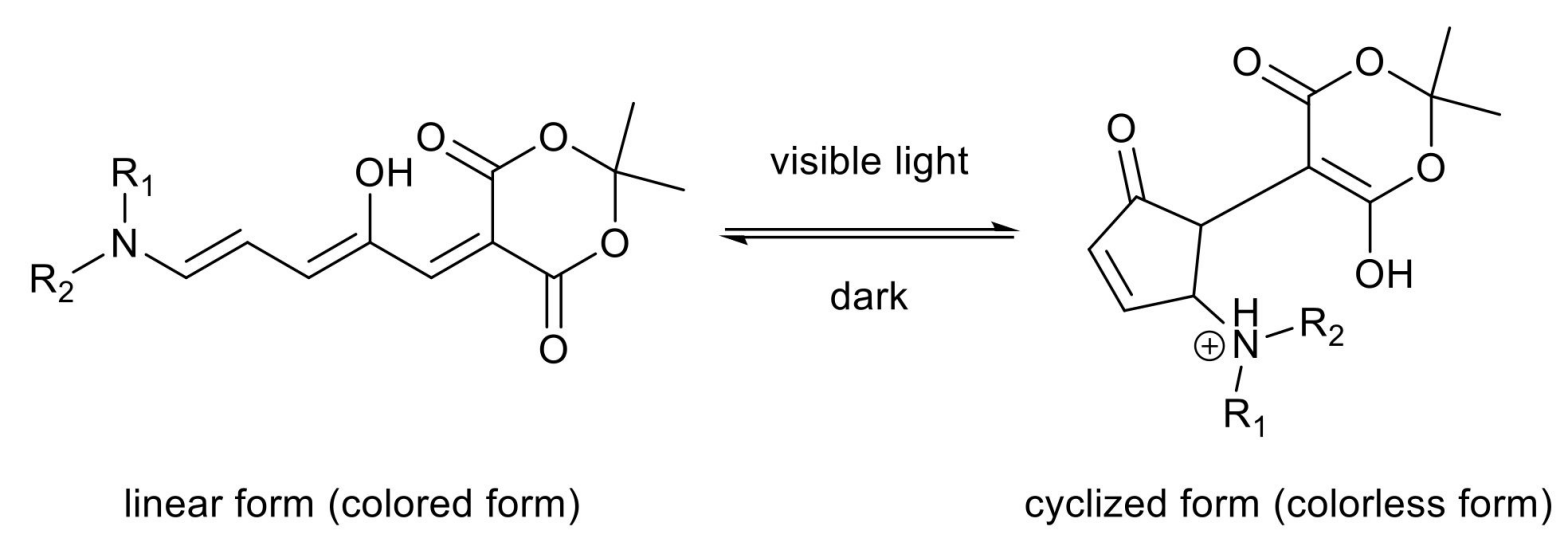
General structures of the open/closed forms of the Donor–acceptor Stenhouse adducts (DASA) based on Meldrum’s acid.

In 2014, a new class of photochrome dyes was developed by Read de Alaniz and coworkers, exhibiting an opposite behavior [19]. Hence, these highly colored molecules, i.e., the Donor-acceptor Stenhouse Adducts (DASA) can be discolored upon photoexcitation with visible light, constituting an original class of negative molecular photoswitches [20]. When kept in the dark, the colored form can be restored by isomerization, the cyclized form being converted into a linear nonpolar form (see Scheme 1). However, DASA are not the only negative photochromes to be reported in the literature and bistable hydrazones have been discovered concomitantly to DASA [21,22,23,24,25,26,27,28,29,30,31,32,33].

Since 2014, several applications have been found for these molecular photoswitches such as the development of light-responsive drug delivery systems [34,35], liquid crystals [36], or colorimetric sensors [37,38]. To the best of our knowledge, DASA have never been reported as polymerization photoinitiators, despite the fact their strong absorption in the visible region makes these dyes ideal candidates for such an application. Indeed, compared to the traditional UV photoinitiators, highly colored molecules constitute appealing candidates for the elaboration of visible light photoinitiating systems which are currently actively coveted for their significant advantages including irradiation safety, curing depth and equipment cost [39,40,41,42,43]. Notably, by developing dyes with high molar extinction coefficients, the photoinitiator content can be drastically reduced while maintaining an efficient production of radicals. Another point governing the reactivity of photoinitiators is also the light penetration inside the photocurable resin. Indeed, if the light penetration is limited to a few hundreds of micrometers at 400 nm, this latter can reach 5 cm at 800 nm, clearly evidencing the benefits to develop photoinitiating systems activable in the near-infrared region [44]. To end, DASA are chromophores well-known to exhibit high molar extinction coefficients [45,46,47,48], so their content in the photocurable resin can be drastically reduced, addressing the photoinitiator extractability issue [48,49,50,51,52].

**Figure 1:**
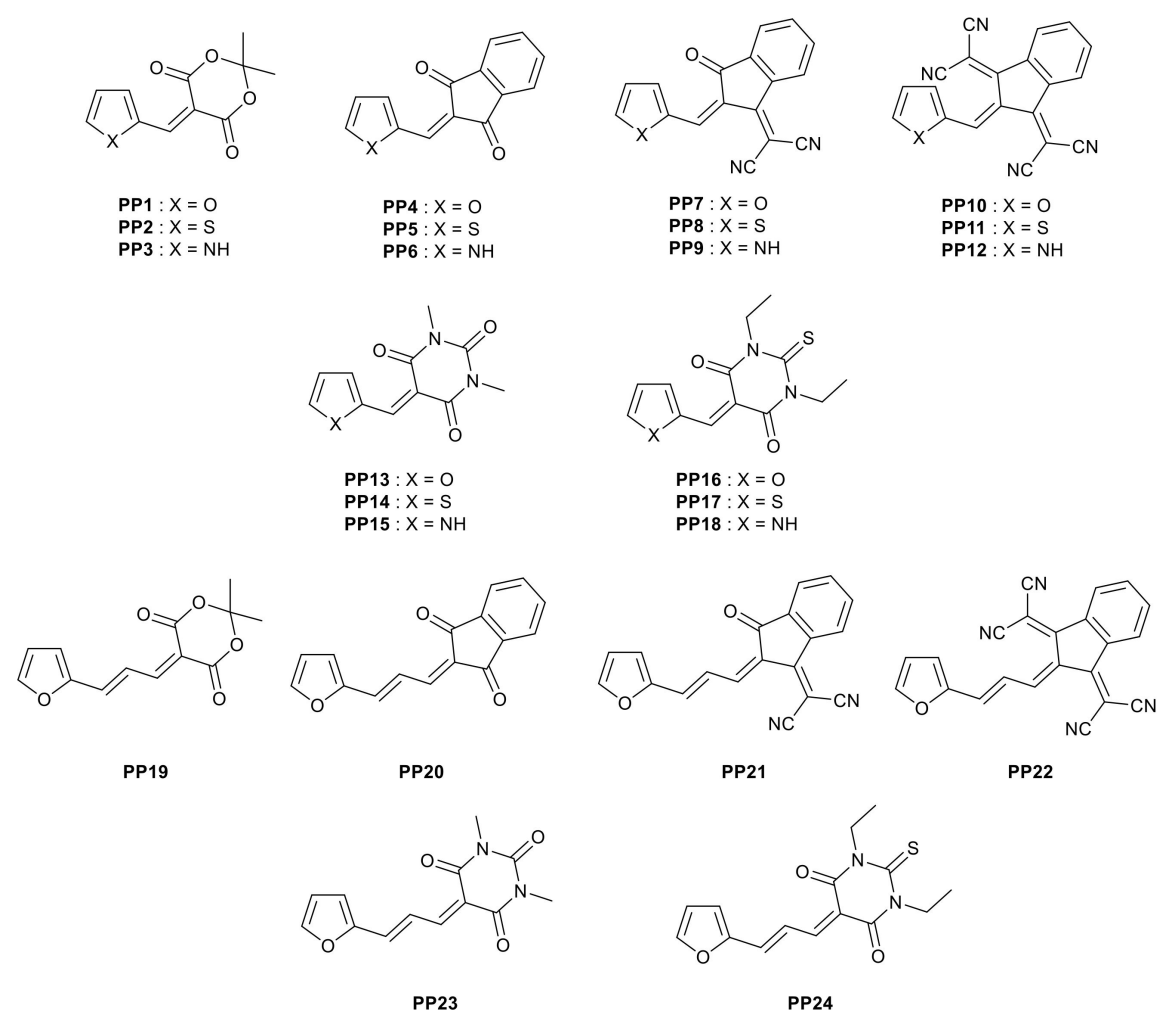
Chemical structures of **PP1**–**PP24** used as precursors for the design of Donor–acceptor Stenhouse adducts (DASA) photoswitches.

**Figure 2:**
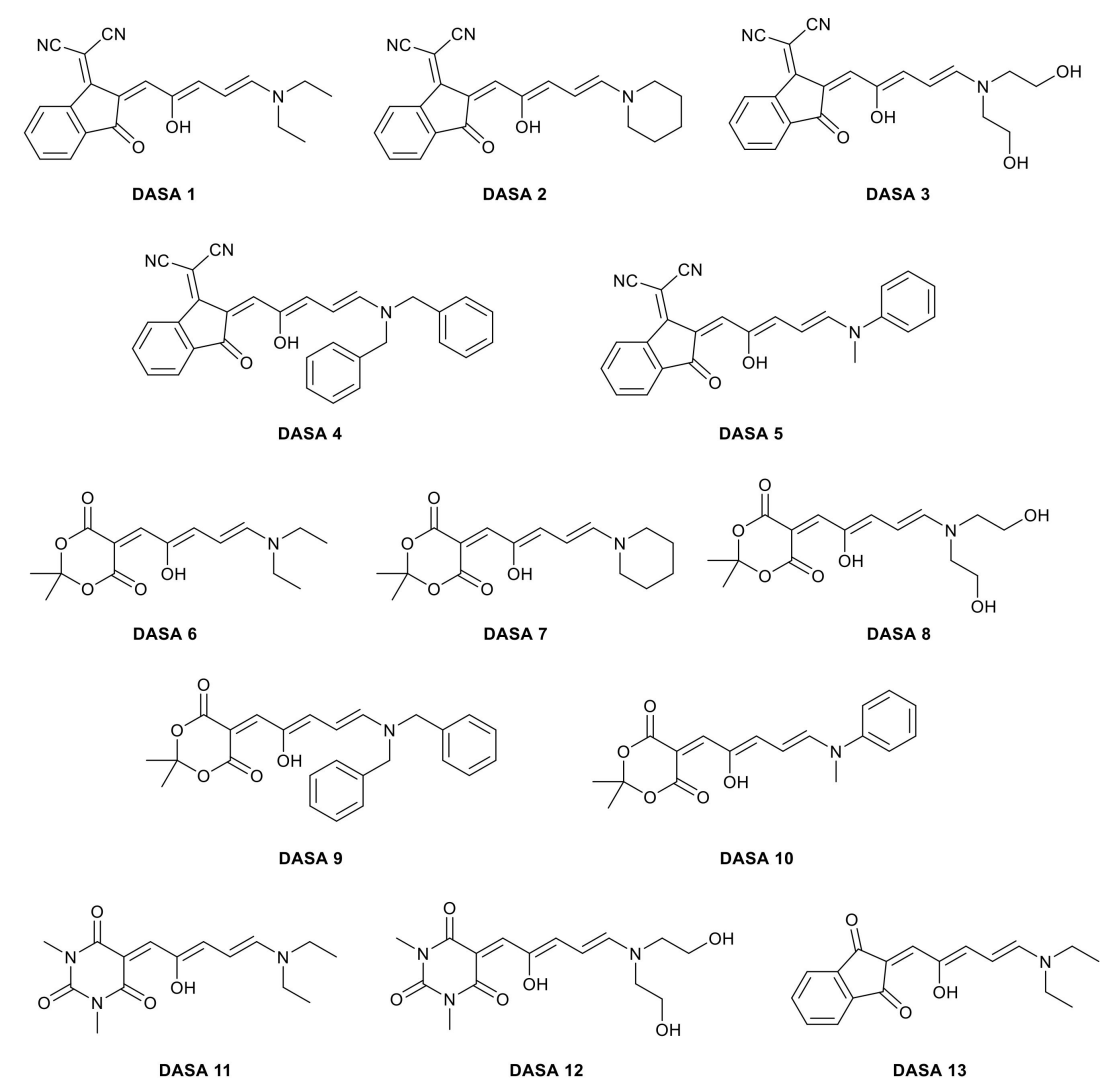

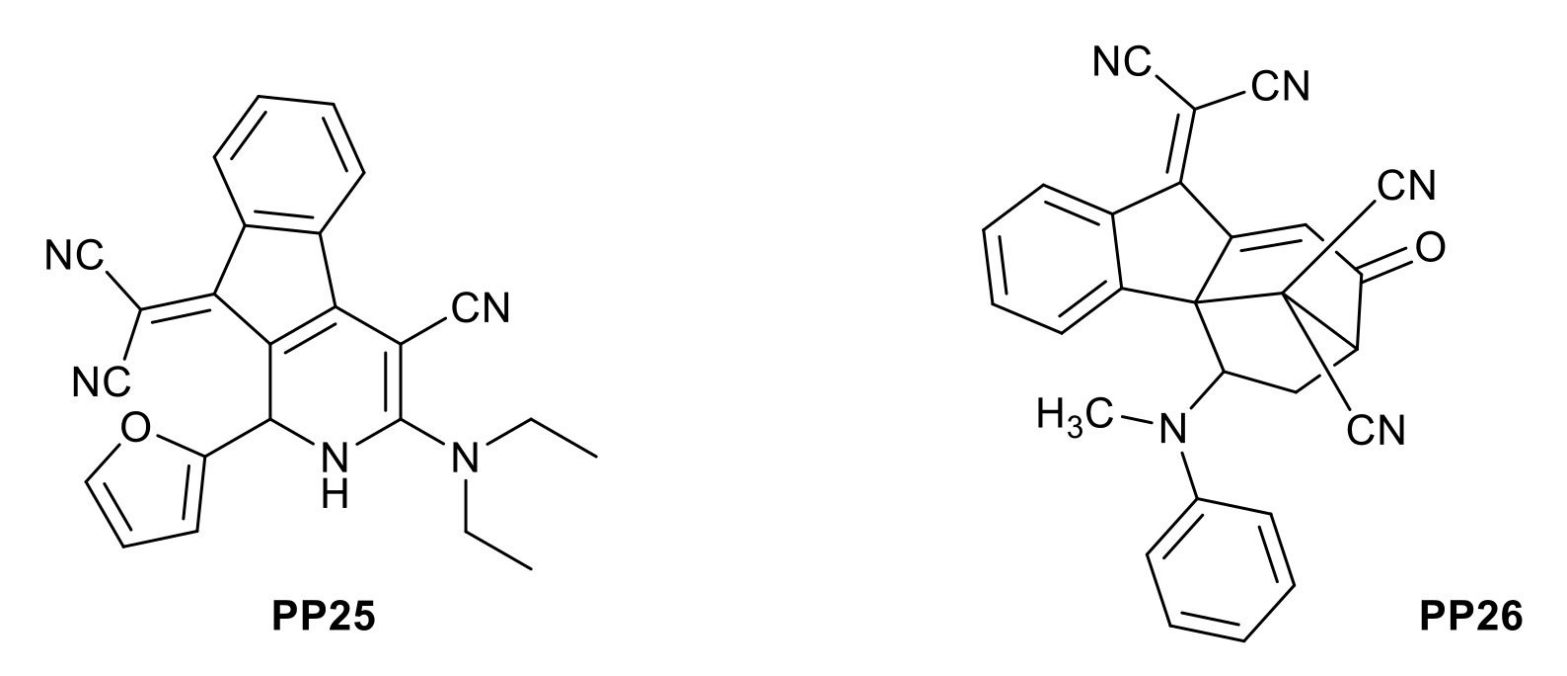
Chemical structures of **DASA 1**–**DASA 13**, **PP25** and **PP26**.

Facing these different considerations, in this work, a series of twenty-four push-pull adducts **PP1**–**PP24** which represent precursors for the design of DASA photoswitches have been examined as potential precursors for the synthesis of Stenhouse adducts (see Figure 1). Parallel to this, several DASA photoswitches (**DASA1**–**DASA13**) have been prepared and examined as polymerization photoinitiators activable in the near infrared region. Interestingly, during the course of our investigations, some unexpected reactions occurred during the synthesis of DASA (see Figure 2) and the cyclization products **PP25** and **PP26** obtained during these reactions have been identified and also tested as polymerization photoinitiators.

## 2. Results and Discussion

### 2.1. Synthesis of ***PP1**–**PP24***

**Scheme 2:**
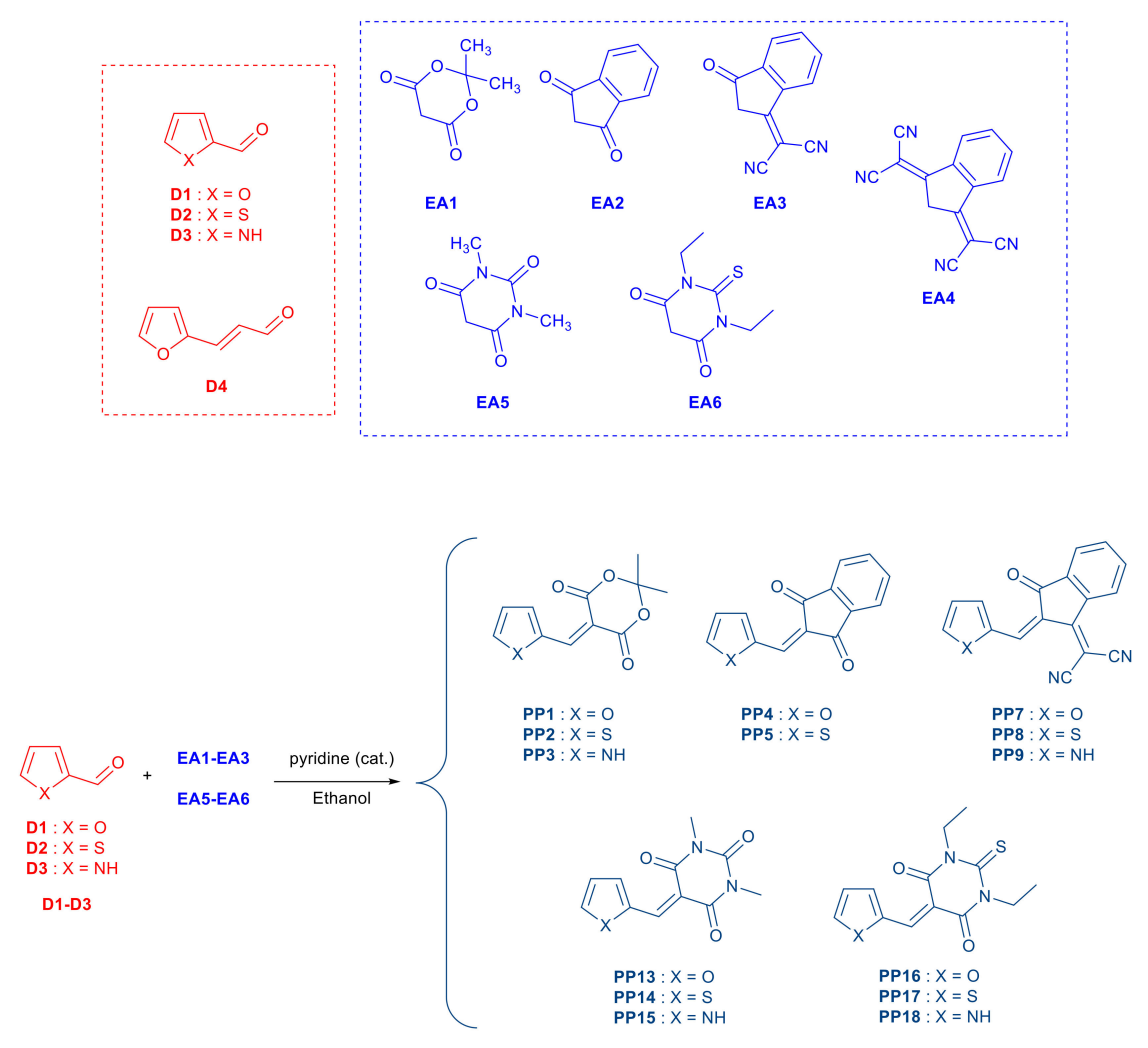

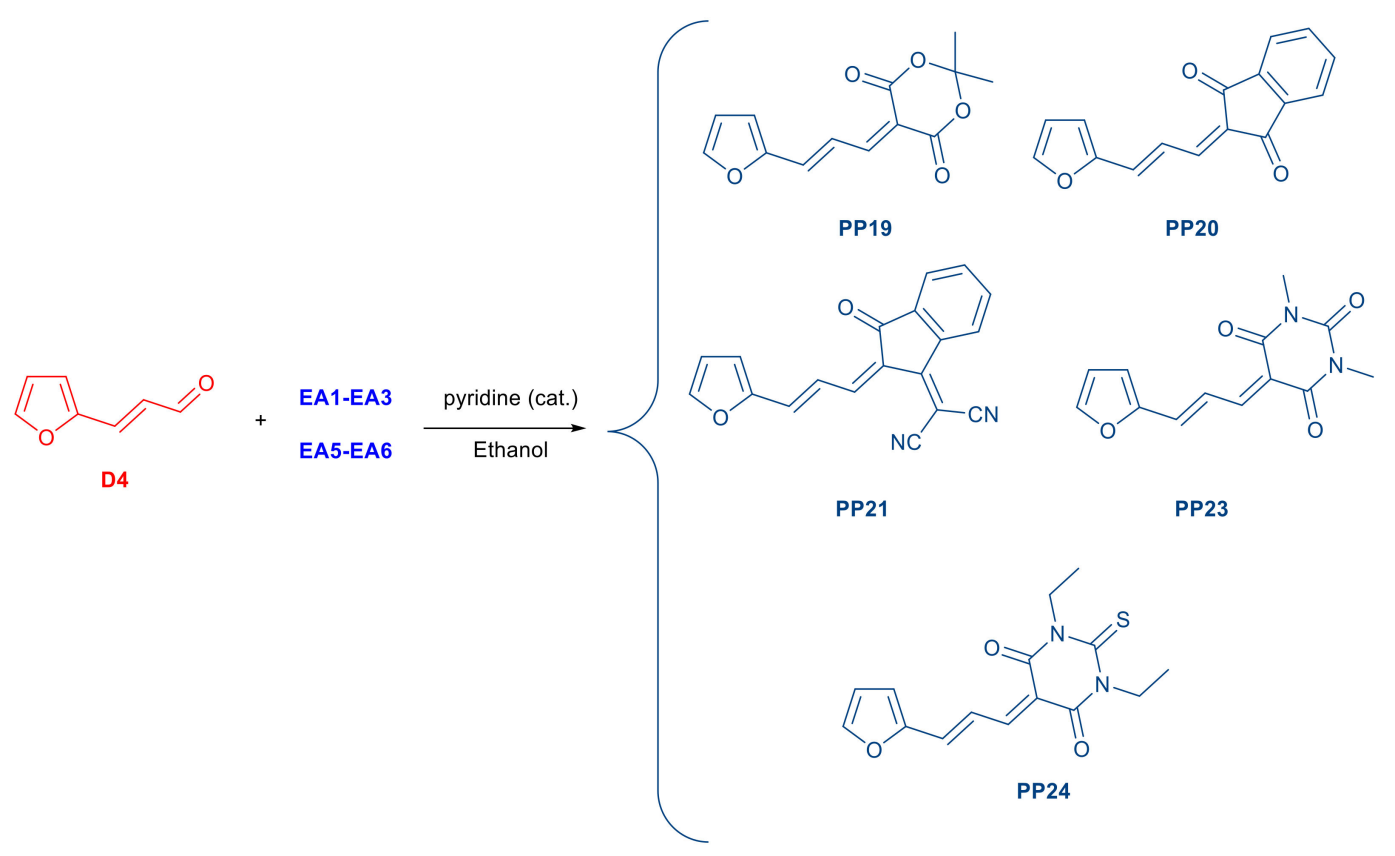
Synthetic route to **PP1**–**PP5**, **PP7**–**PP9**, **PP13**–**PP21**, **PP23**–**PP24**.

**PP1**–**PP24** were prepared by a Knoevenagel reaction combining four electron donors **D1**–**D4** with six different electrons acceptors **EA1**–**EA3**, **EA5**–**EA6**. Considering that piperidine can initiate an undesired nucleophilic addition on the push-pull dyes subsequent to their syntheses, pyridine was selected as the base to catalyze the Knoevenagel reaction (see Scheme 2). Synthetic procedures are detailed in the Supplementary Materials.

**Scheme 3:**
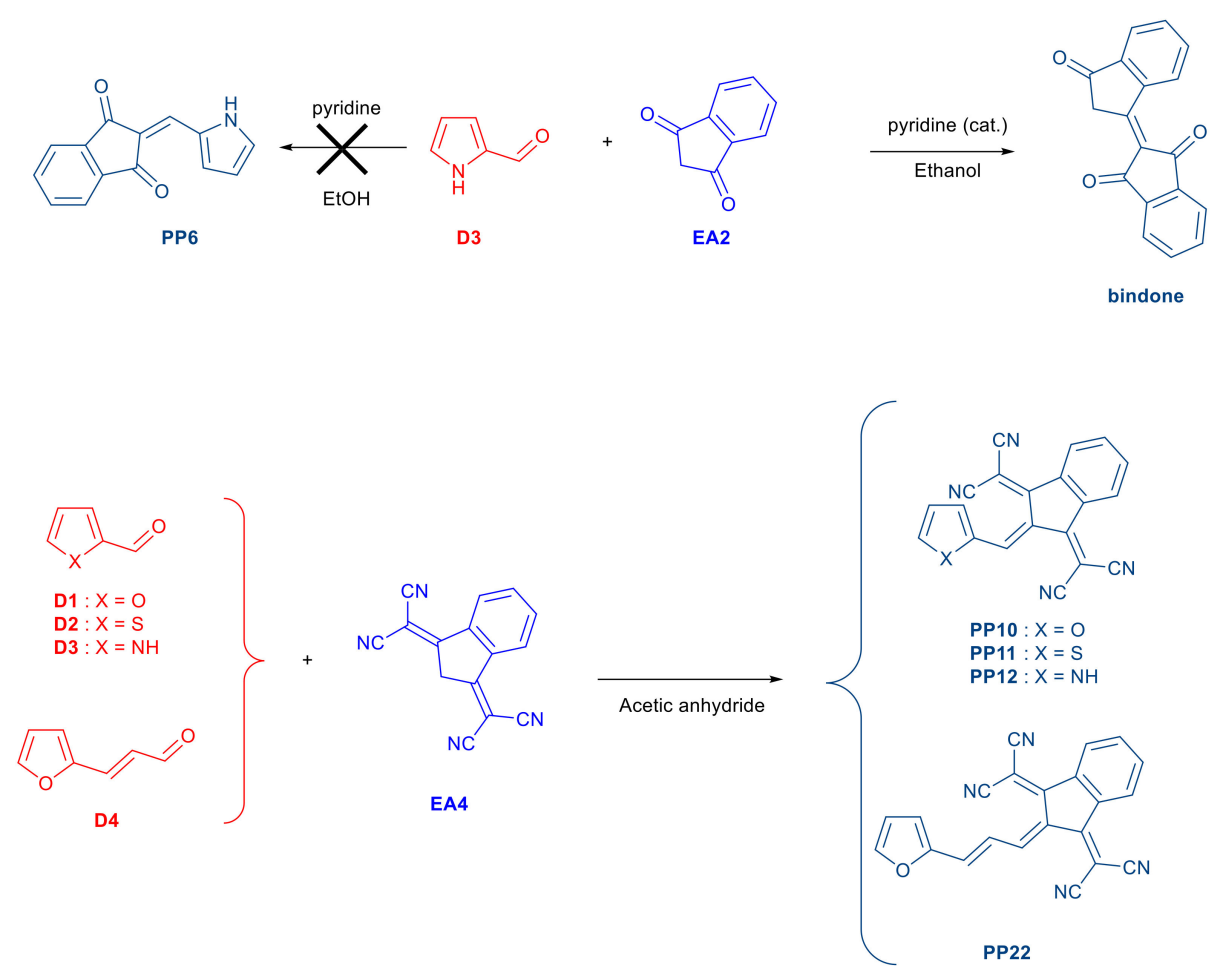
Synthetic route to **PP10**–**PP12** and **PP22**.

All dyes, except for **PP6**, that could not be synthesized, were obtained in good yields ranging from 79% for **PP24** to 97% for **PP16** (see Table 1). Indeed, instead of **PP6**, [1,2′-biindenylidene]-1′,3,3′(2*H*)-trione, also named bindone and resulting from the self-condensation of indane-1,3-dione, was isolated in 89% yield as the sole reaction product (see Scheme 3).

This is directly related to the low reactivity of pyrrole-2-carboxaldehyde **D3**, favoring the dimerization of indane-1,3-dione **EA2**. Concerning the synthesis of **PP10**–**PP12** and **PP22** with **EA4**, a completely different synthetic route had to be used due the remarkable stability of the **EA4** anion under basic conditions. To overcome this drawback, the Knoevenagel reactions were carried out in acetic anhydride and **PP10**–**PP12** and **PP22** could be isolated with reaction yields ranging from 82% to 88% yield (see Scheme 2 and Table 1).

### 2.2. Synthesis of ***DASA1***–***DASA13*** and Cyclized Structures ***PP25*** and ***PP26***

**Scheme 4:**
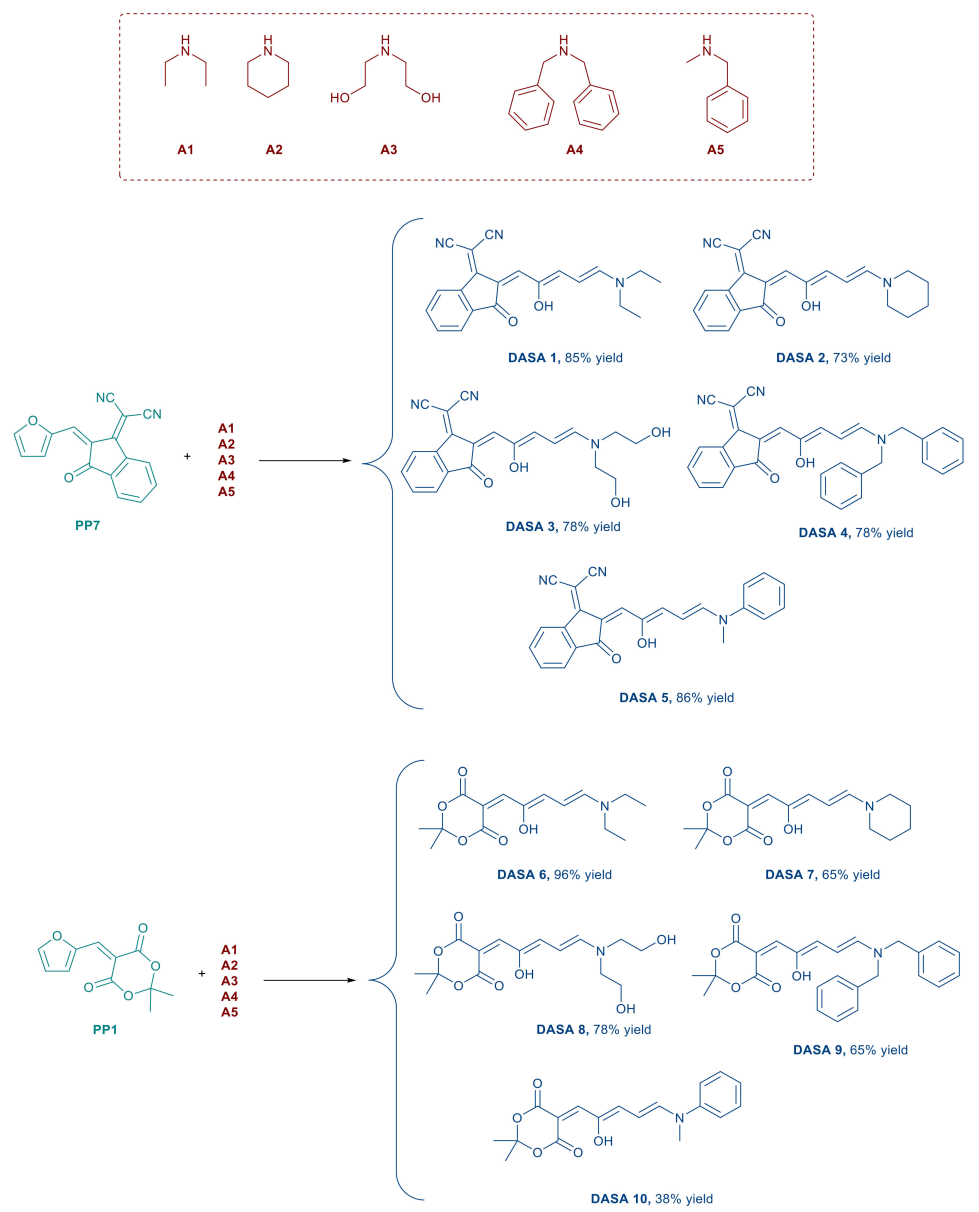
Synthetic route to **DASA1**–**DASA10**. Investigated amines were: diethylamine (**A1**), piperidine (**A2**), diethanolamine (**A3**), dibenzylamine (**A4**) and *N*-methylbenzylamine (**A5**).

**Scheme 5:**
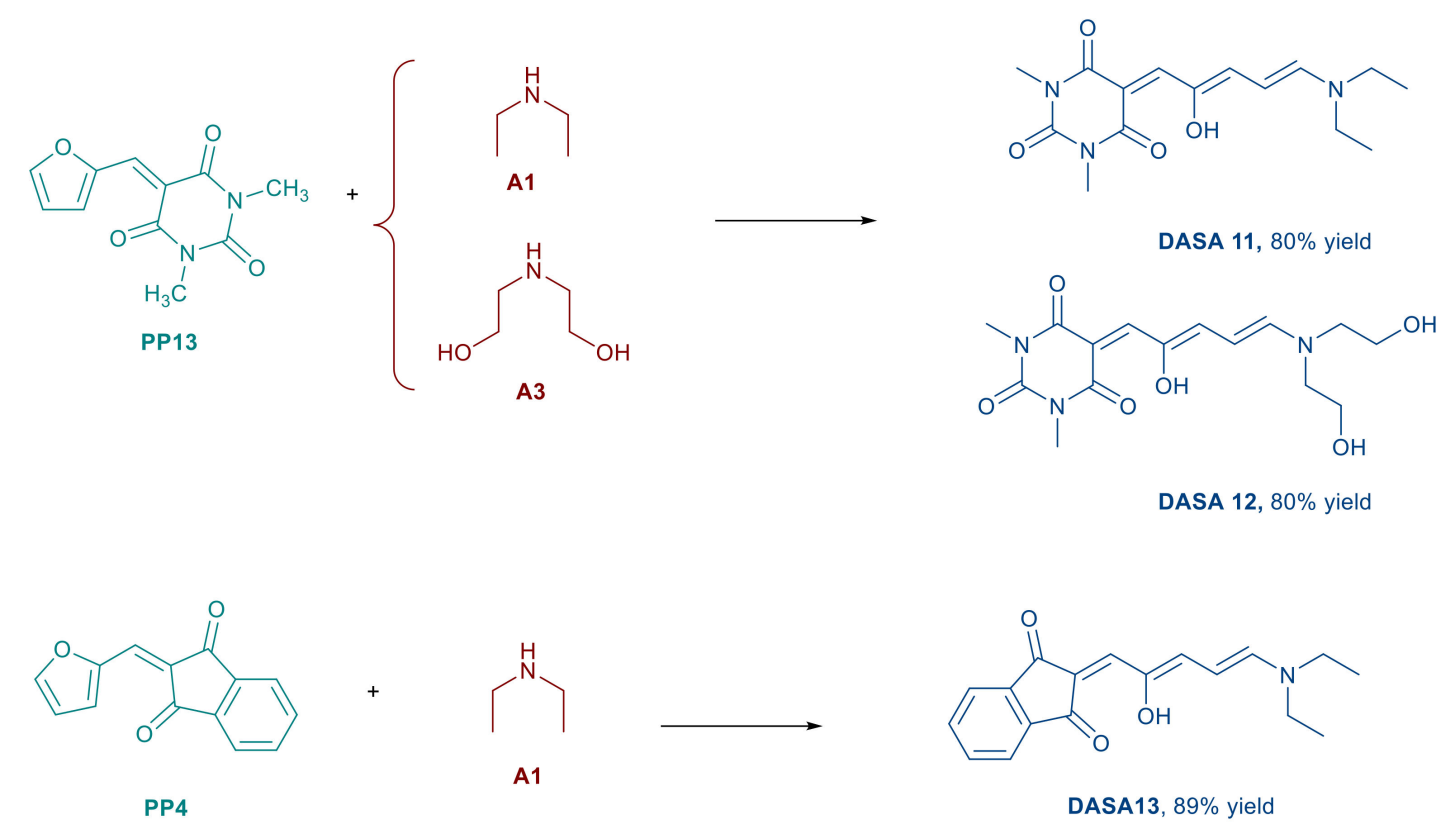
Synthetic route to **DASA10**–**DASA13**.

**Scheme 6:**
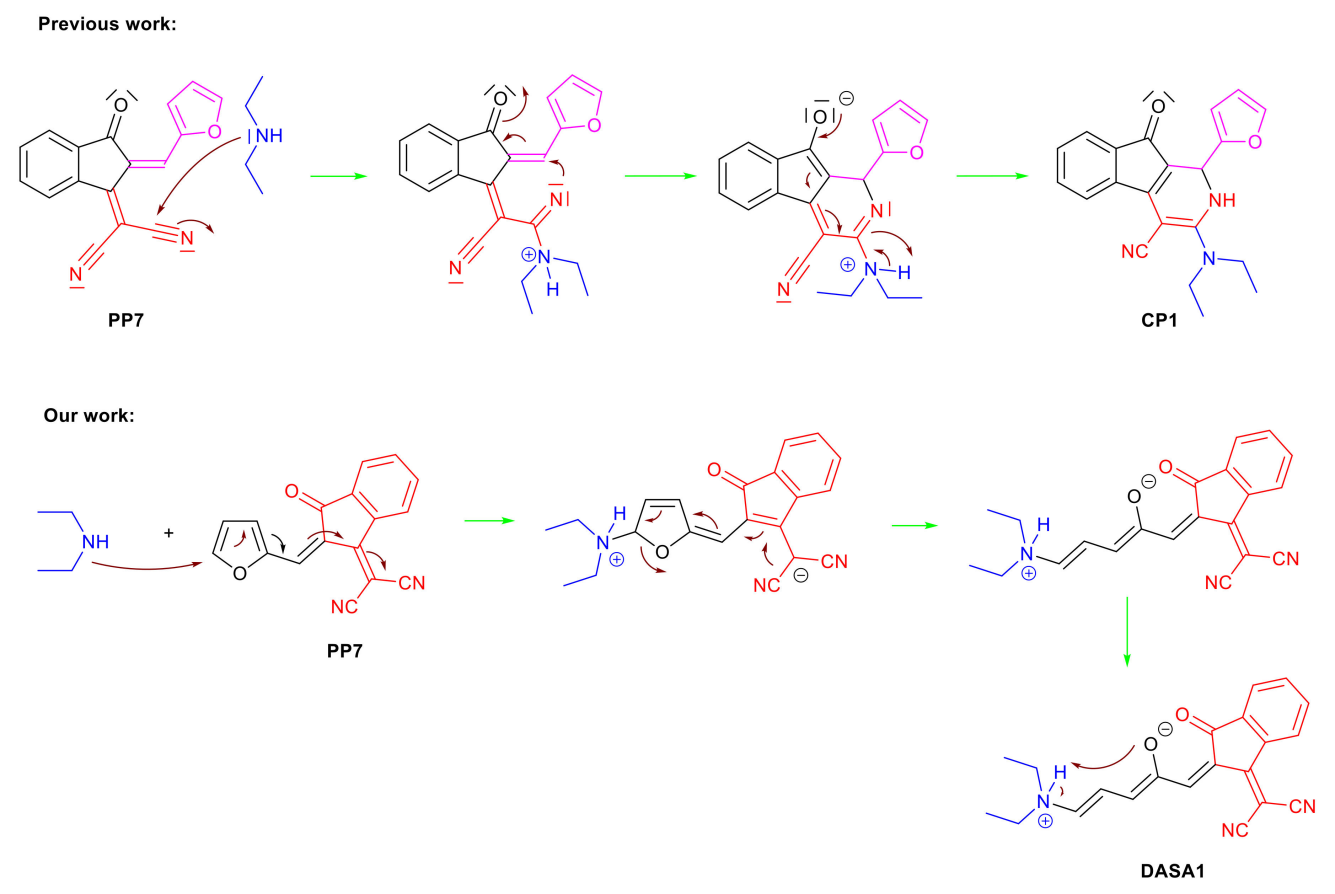
Mechanisms involved in the synthesis of cyclization product (**CP1**) and **DASA1**.

For the synthesis of the Stenhouse adducts, five different amines were investigated, namely, diethylamine (**A1**), piperidine (**A2**), diethanolamine (**A3**), dibenzylamine (**A4**), and *N*-methyl-benzylamine (**A5**). When examining the nucleophilic addition of these five amines **A1**–**A5** to **PP1**–**PP20**, no reaction was detected for any of the dyes bearing thiophenes or pyrroles as heterocycles. This is directly related to an aromaticity increase of the order furan < pyrrole < thiophene, impeding the ring-opening reaction of pyrrole and thiophene by a nucleophile [53,54,55]. A similar behavior was also observed with **PP17**–**PP22**, despite the presence of the furyl group. In this case, no ring opening reaction could occur with the different amines due to the elongation of the spacer introduced between the furyl donor and the acceptors. As a result of this, the electron-withdrawing ability of the different acceptors was insufficient to promote the ring-opening reaction. Conversely, as expected, the nucleophilic addition of the different amines to **PP1**, **PP4**, **PP7** and **PP13** could proceed, promoting the synthesis of the expected Stenhouse adducts in high yields (see Scheme 4, Scheme 5 and Scheme 6).

Notably, reaction yields ranging between 38% for **DASA10** and 96% for **DASA6** could be obtained. It should be noted that several of the Stenhouse adducts could not be obtained and our results are consistent with those previously reported in the literature [19]. One exception was noted, that of **DASA1**. Indeed, **DASA1** could be prepared in 85% yield whereas in previous investigations [19] its synthesis was reported as being impossible. When using the same reaction conditions used with **PP7** and diethylamine, Read de Alaniz and coworkers reported a nucleophilic attack of piperidine on the cyano groups of **PP7**, producing the cyclization product **CP1** in 96% yield as the only reaction product according to the mechanism presented in Scheme 6.

**Scheme 7:**
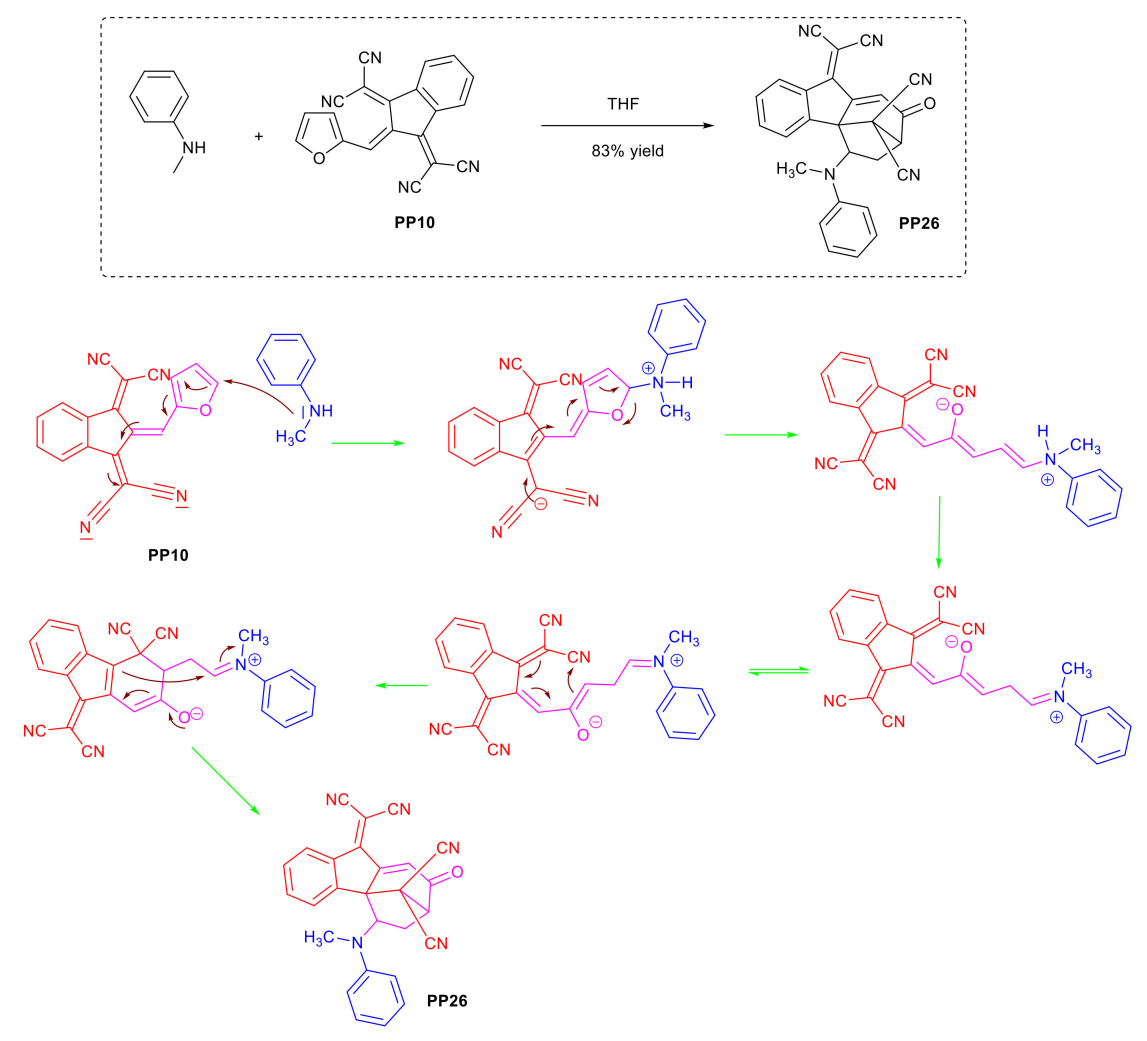
Mechanisms supporting the formation of **PP26**.

It should be noticed that the nucleophilic addition of secondary amines onto the cyano groups of push-pull dyes has been recently reported in the literature as a side-reaction occurring during the synthesis of push-pull dyes comprising **EA3** or **EA4** as the electron acceptors [56,57,58,59,60]. The preferential nucleophilic addition of diethylamine on cyano groups rather than onto the electrodeficient furyl group was confirmed during the synthesis of **PP25** starting from **PP7** and diethylamine (**A1**). **PP25** could be isolated in pure form in 89% yield. Unexpectedly, a more complex reaction occurred when **PP10** was reacted with *N*-methylbenzylamine (**A5**) as the nucleophile. As shown in Scheme 7, the nucleophilic addition of compound **A5** occurred on the furyl side, as classically observed during the synthesis of the Stenhouse adduct. By an addition/elimination mechanism, a ring-opening of the furyl group can occur. Following a proton transfer, an iminium cation is formed. By a 6π electrocyclization, a six-membered ring can then be obtained. Finally, a nucleophilic attack on the iminium cation furnishes **PP26** as a polycyclic molecule. It should be mentioned that such a cascade reaction is unprecedented in the literature (See Scheme 7).

**Figure 3:**
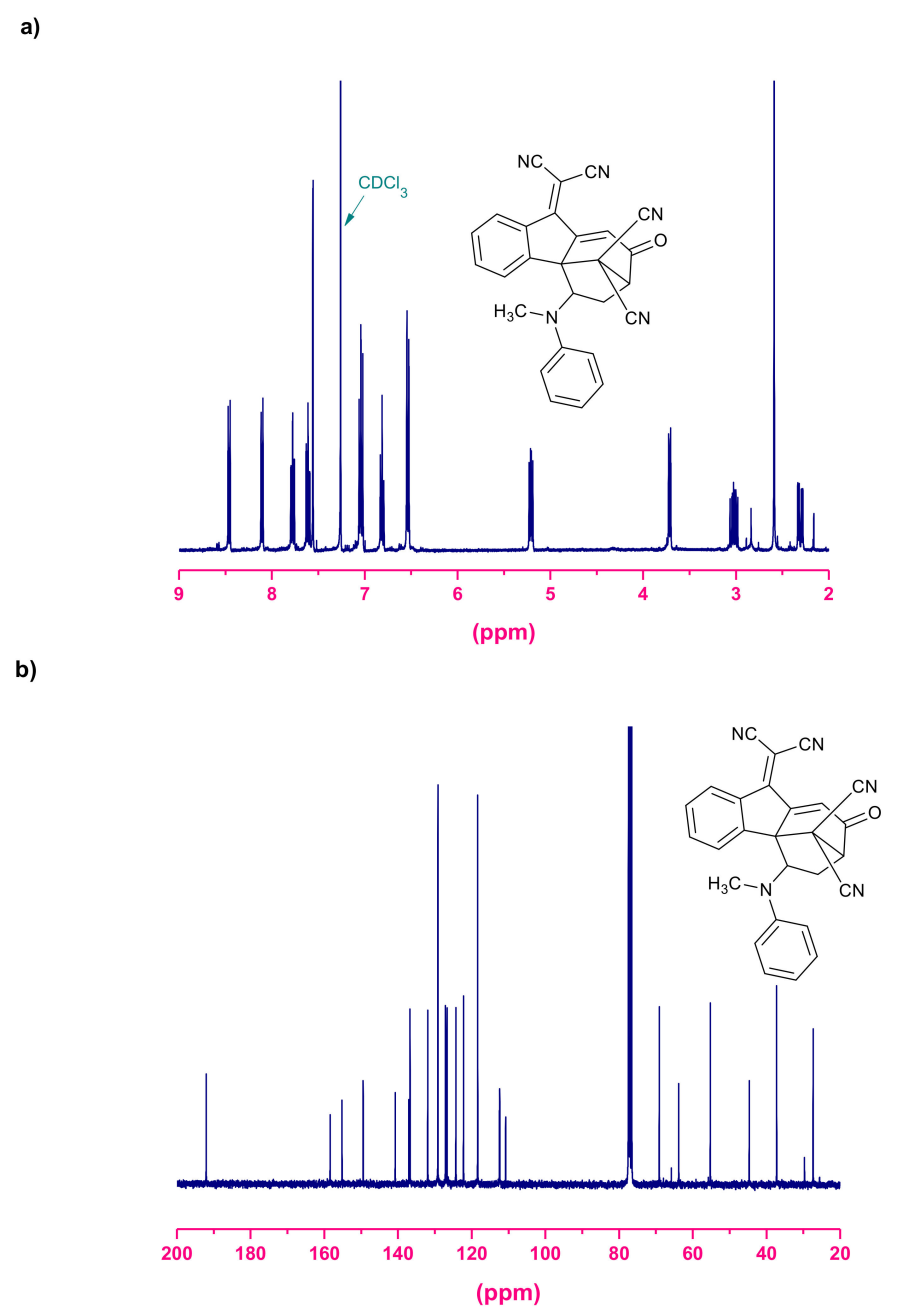
Proton nuclear magnetic resonance (^1^H-NMR) spectrum (**a**) and carbon-13 nuclear magnetic resonance (^13^C NMR) spectrum (**b**) of cyclized compound **PP26**.

The chemical structure of **PP26** was confirmed by proton (^1^H) and carbon-13 nuclear magnetic resonance (^13^C-NMR) analyses(Avance 400 spectrometer, Bruker, Billerica, MA, USA), as detailed in the Figure 3. Notably, in the 2.0–5.5 ppm region, the four aliphatic protons in the cycles of **PP26** as well as the methyl group of *N*-methylbenzylamine can be detected in the ^1^H-NMR spectrum. The presence of aliphatic protons in the chemical structure of **PP26** was confirmed by ^13^C-NMR analyses, where six carbons could be detected between 20 and 70 ppm.

**Figure 4:**
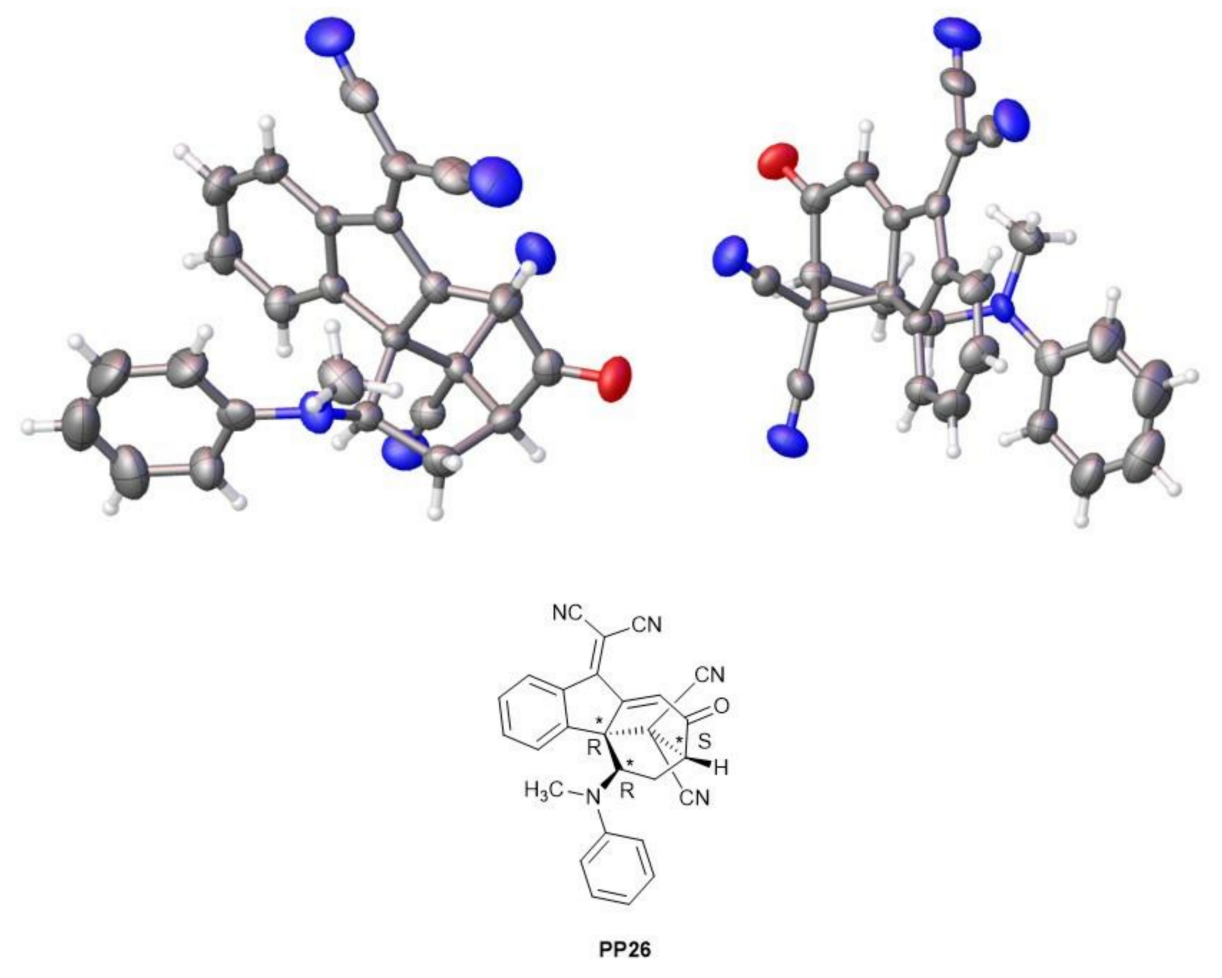
Crystal structure of **PP26**.

Finally, by slow evaporation of the deuterated solvent in an NMR tube, crystals of **PP26** could be obtained and the crystal structure was resolved by X-ray crystallography (see Figure 4). It should be noted that only one of the possible diastereoisomers has been found in the crystals, despite the analysis of several crystals to confirm this point. Based on the crystal structure, the relative stereochemistry of **PP26** could be determined as (4*R*,5*R*,7*S*)-10-(dicyanomethylene)-5-(methyl-(phenyl)amino)-8-oxo-6,7,8,10-tetrahydro-5*H*-4*b*,7-methanobenzo[*a*]azulene-11,11-dicarbonitrile (see Figure 4). Configurations of the asymmetric carbons can also be found in the Checkcif. As also evidenced in the ^1^H- and ^13^C-NMR spectra of **PP26** (see Figure 3, and details concerning the crystal structure of **PP26** can be found in the Supplementary Materials), only slight traces of diastereoisomers could be detected on the baseline, at 7.15 and 8.62 ppm, respectively.

### 2.3. Photopolymerization Experiments

**Figure 5:**
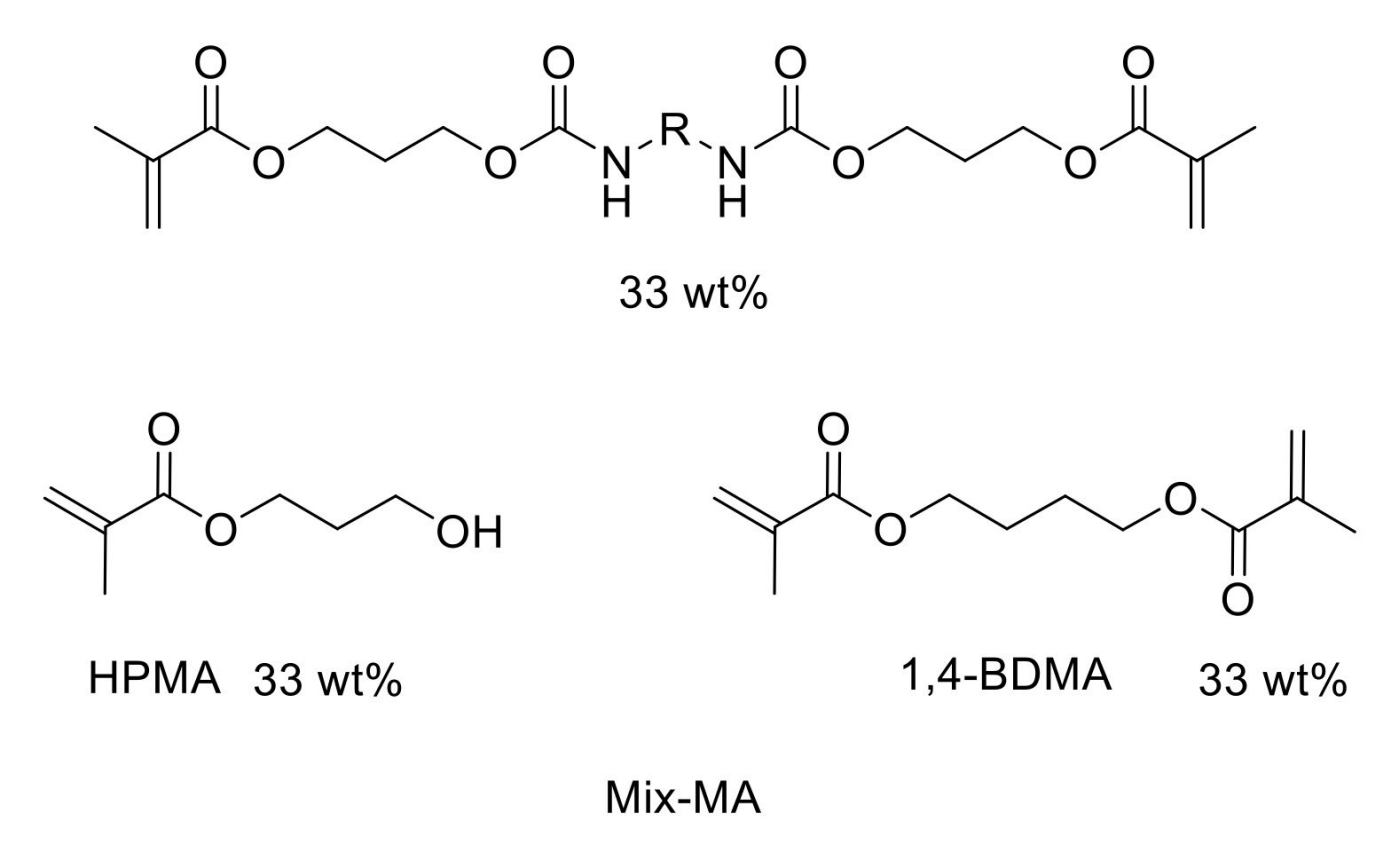
Benchmark methacrylate monomer blend Mix-MA.

A benchmarked methacrylate monomer blend, “Mix-MA” was used here, the composition of which is presented in Figure 5. This blend has been prepared with 33.3 *wt*% of (hydroxypropyl)methacrylate (HPMA), 33.3 *wt*% of 1,4-butanediol dimethacrylate (1,4-BDMA) and 33.3 *wt*% of a urethane dimethacrylate monomer (UDMA), obtained from Sigma Aldrich (St. Louis, MO, USA). This benchmark blend has an adapted viscosity for polymerization under air [44,61,62,63,64].

**Figure 6:**
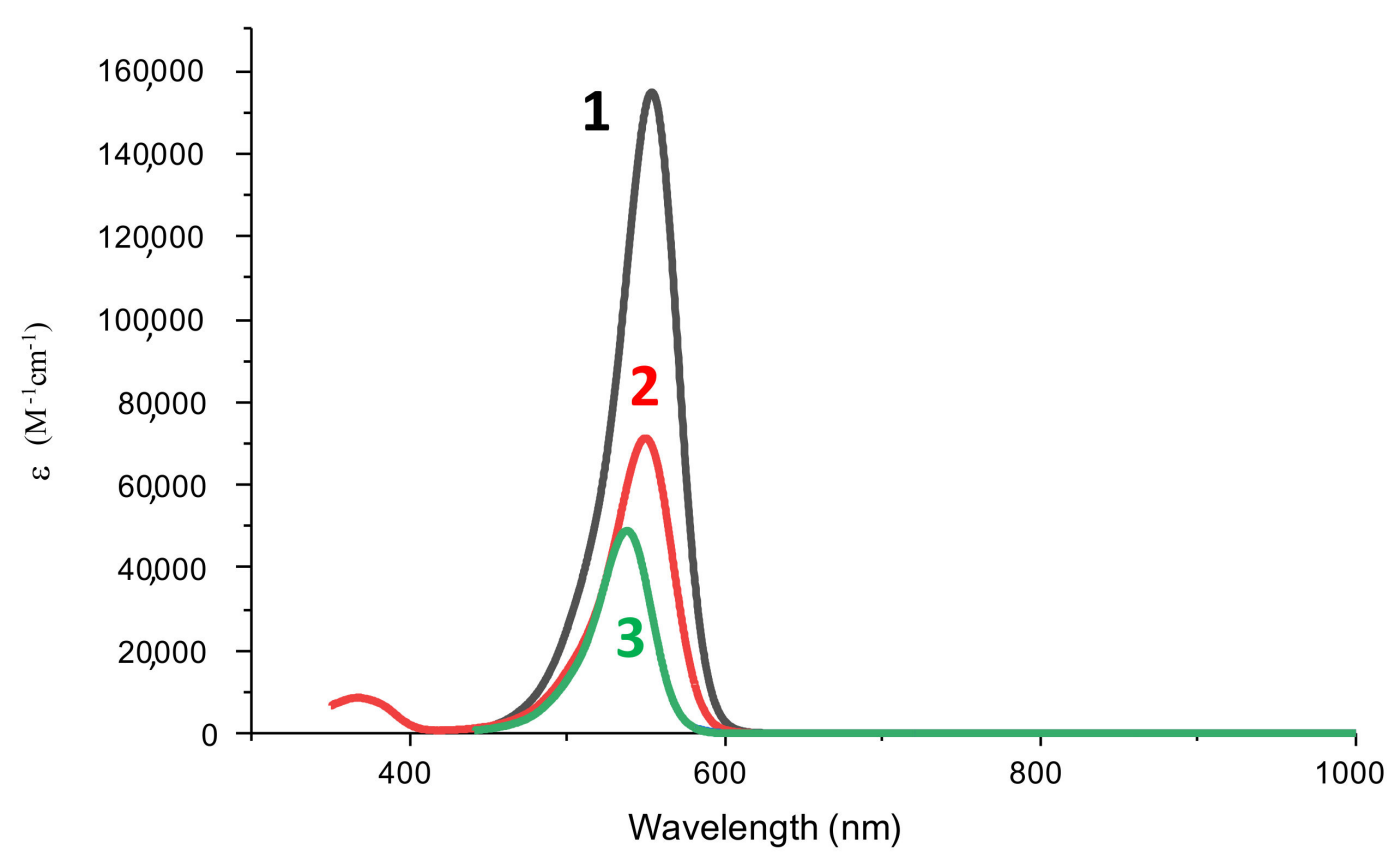
UV-vis absorption spectra of **DASA12** (1); **DASA11** (2) and **DASA9** (3) in acetonitrile.

It has to be noticed that the 13 **DASA** dyes **DASA1**–**DASA13** examined in this work are Stenhouse adducts meaning that these structures can discolor upon irradiation in the visible range. However, the efficiency of the discoloration process is directly related not only to the excitation wavelength, but also the intensity of the light source [19]. In the present case, 785 nm was selected as the wavelength for photopolymerization due to the weak absorption of the different Stenhouse adducts in this region, ensuring slow cyclization kinetics with regards to the photopolymerization timescale. The optical density at 785 nm for these 1.4 mm samples is always < 0.1 to ensure a good light depth penetration (any inner filter effect must be avoided for polymerization in depth). Indeed, these dyes are characterized by low extinction coefficients at 785 nm (<20 M^−1^ cm^−1^) that ensure such low absorption at 785 nm. The absorption of DASA dyes is mainly centered in the 500–600 nm range (see Figure 5) with extremely high molar extinction coefficients. However, polymerization cannot be efficiently initiated in this latter spectral range due to the low efficiency of photothermal processes in the 500–600 nm range (less efficient than in the near-infrared (NIR) region, see Figure 6).

**Figure 7:**
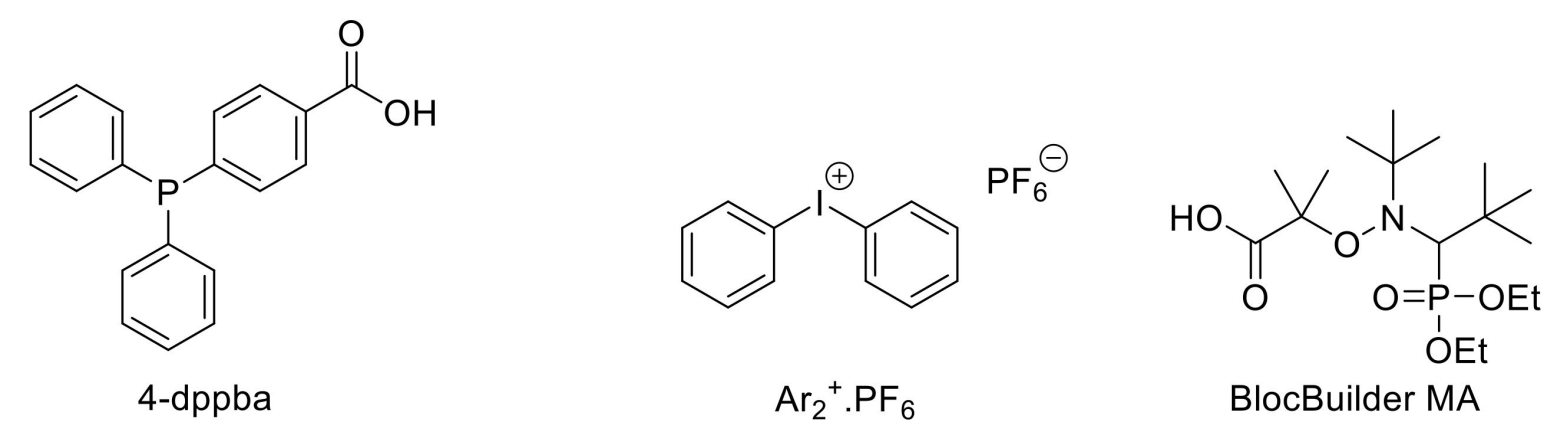
Chemical structures of additives.

Additionally, all formulations were kept in the dark prior to photopolymerization to ensure the different dyes would be in their colored forms. To perform the irradiation at 785 nm, a NIR laser diode with a tunable irradiance from 0 W to 2.55 W/cm² was purchased from Changchun New Industries (CNI, Changchun, China) and used here. The kinetics of photopolymerization of the photosensitive resins were followed through the double bond C=C conversion vs. time upon NIR irradiation (see Figure 7). The peak followed by Real Time Fourier Transform Infrared (RT-FTIR) spectroscopy (using a Jasco 4600 instrument, Jasco, Tokyo, Japan) is located at 6100–6220 cm^−1^ as presented in [37]; this peak corresponds to the first overtone of the C-H vibrations of the =CH_2_ acrylate group.

The NIR photoinitiating systems are composed of (NIR) dyes (**DASA1**–**DASA13**, **PP25** or **PP26**) combined with an iodonium salt (Ar_2_I^+^.PF_6_), a phosphine (4-dppba) and a thermal initiator (here BlocBuilder MA) (see Figure 7) to take advantage of the photoinitiating system (NIR dye/iodonium/phosphine) associated with the photothermal system (NIR dye/BlocBuilder MA) as presented for other NIR dyes in [64].

**Figure 8:**
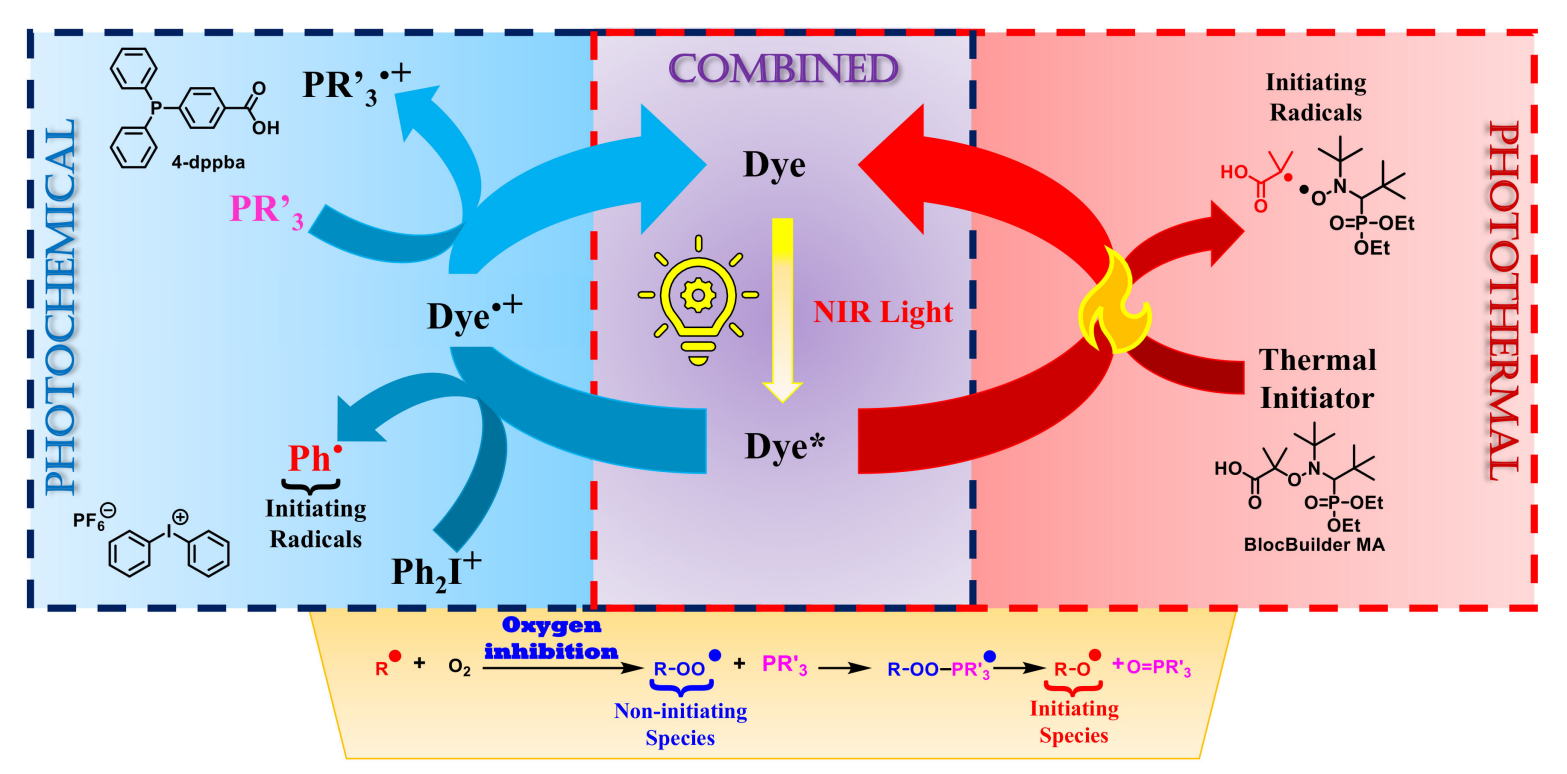
Overview of the chemical mechanism.

The expected mechanisms are given in r1–r5. The free radicals are generated through two different mechanisms: (1) a photochemical pathway through the interaction of the dye with the iodonium salt i.e., the photoreduction of the iodonium salt by the excited state of the dye leads to the formation of aryl radicals that are very efficient to initiate polymerization processes (r1) and (2) a photothermal effect i.e., the dye is able to convert NIR light into heat that decomposes the thermal initiator (r2–r3). For the activation 1 and 2, the phosphine is used to overcome the oxygen inhibition as all the polymerization processes were carried out under air through r4–r5 i.e., the phosphine is able to convert non-initiating peroxyls (ROO^●^) to reactive alkoxyl radicals (RO^●^) [40]. All these chemical mechanisms can also be depicted by Figure 8. Overall, by combining a photochemical and a thermal polymerization process, four distinct types of radicals can potentially be formed, namely, Ar^●^, HOOC-C^●^(Me)_2_ as the primary radical sources and to a less extent, the radicals resulting from the decomposition of the non-initiating peroxyls by 4-dppba i.e., PhO^●^ and HOOC-CO^●^(Me)_2_. It has to be noticed that the autooxydation of triarylphosphine by peroxy radicals has been extensively studied by Buckler and coworkers in 1962. In his work, he suggested the oxidation of the triarylphosphine to occur via a radical attack on the heteroatom, resulting in a valence expansion producing a short-lived phosphoranyl radical. By mean of a β-scission, a phosphine oxide can be formed due to the weak O-O bond [65,66]. More recently, exact role of phosphines in photopolymerization processes has been examined by the group of Lalevée et al. [67].

dye → *dye (hν)

*dye + Ar_2_I^+^ → dye^●+^ + Ar^●^ + ArI (r1)

*dye → dye + ∆(r2)

Blocbuilder + ∆ → free radicals(r3)

ROO^●^ + PAr_3_ → ROO-P^●^Ar_3_ (r4)

ROO-P^●^Ar_3_ → RO^●^ + O=PAr_3_ (r5)

**Figure 9:**
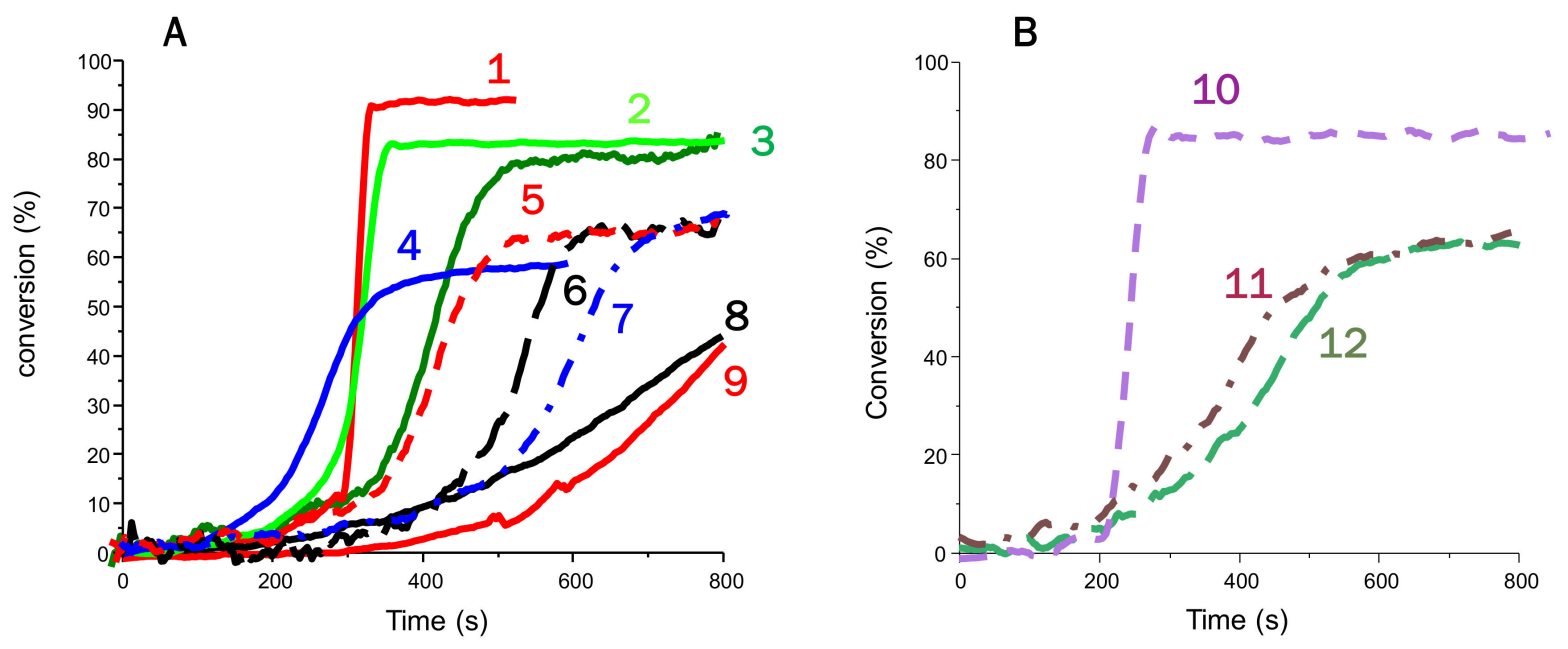
Real Time Fourier Transform Infrared (RT-FTIR) photopolymerization kinetics (C=C conversion vs. irradiation time; 1.4 mm thick sample) under air upon exposure to a Laser Diode@785nm; 2.5 W/cm²; for Mix-MA in the presence of dye/iodonium/4-dppba/BlocBuilder MA(0.1/2/2/2% *w/w*); the irradiation starts after 15 s for: (**A**) (1) **DASA1**; (2) **DASA2**; (3) **DASA3**; (4) **DASA4**; (5) **DASA5**; (6) **DASA12**; (7) **DASA10**; (8) **DASA9**; (9) **DASA11**; (**B**) (10) **DASA7**; (11) **PP25**; (12) **PP26**.

Interestingly, twelve of the investigated structures exhibit significant polymerization processes upon NIR light at 785 nm under air (final methacrylate function conversion (FC) > 20%) (see Figure 9). For these photopolymerization reactions, no clear bleaching is observed. For the other dyes (**DASA6**, **DASA8** and **DASA13**), rather poor polymerization processes are observed with FC << 20%. Interestingly for **DASA1**, **DASA2** and **DASA7**, fast and efficient polymerization processes are observed despite a rather long lag time of ~200 s ascribed to the oxygen inhibition (i.e., oxygen must be consumed before the radical polymerization can start). This excellent reactivity shows that these DASA dyes can work through combined approach (photochemical and photothermal) to obtain very high final methacrylate function conversion (>80%) in less than 350 s. In reality, only the combined mode gathering photochemical and photothermal activations is really efficient. For the control experiments (only photochemical or photothermal mode), only low conversions are observed (<20%). This shows that only the combined mode leads to a high yield in initiating radicals to overcome the oxygen inhibition. Such a behavior has been found in [64] for other dyes. In this work, only dyes active in this combined mode are efficient.

## 3. Conclusions

In this work, a series of 24 push-pull dyes have been designed as precursors for the synthesis of Stenhouse adducts. Unfortunately, all attempts to prepare Stenhouse adducts with thiophene and pyrrole were unsuccessful due to the high energy of the C-N and C-S bonds. Similarly, all attempts to induce a ring opening reaction of the pyrrole moiety in the **PP19**–**PP24** failed as a result of an insufficient electron-withdrawing ability of the different acceptors upon elongation of the π-conjugated spacer. Finally, 13 DASA adducts could however be prepared and the preliminary tests revealed most of these dyes to be promising candidates as photoinitiators of polymerization for the free radical polymerization (FRP) of acrylates upon excitation at 785 nm. The main parameter governing the efficiency of the dye is its ability to be active both in photothermal and photochemical modes (combined mode). Indeed, only this combined mode is really efficient. Parallel to this, an unexpected cyclization product issued from a cascade mechanism was obtained, resulting from the nucleophilic attack of diethylamine on the furyl group of **PP10**. In light of these preliminary results, no clear trend could be deduced concerning the structure-performance relationship due to the paucity of structures examined. Future works concerning the Stenhouse adducts will consist in examining a broader range of structures in order to establish the full guidelines for the design of highly efficient photoinitiators.

## Figures and Tables

**Table 1:** Reaction yields obtained for the synthesis of **PP1**–**PP24** by Knoevenagel reactions.

**Dyes**	**PP1**	**PP2**	**PP3**	**PP4**	**PP5**	**PP6**	**PP7**	**PP8**
Reaction Yield	85	92	89	72	84	-	83	91
**Dyes**	**PP9**	**PP10**	**PP11**	**PP12**	**PP13**	**PP14**	**PP15**	**PP16**
Reaction Yield	88	83	82	88	89	82	90	97
**Dyes**	**PP17**	**PP18**	**PP19**	**PP20**	**PP21**	**PP22**	**PP23**	**PP24**
Reaction Yield	86	85	87	87	84	81	88	79

## Supplementary Materials

The following are available online, Synthetic procedures of all dyes, Figure S1. Configuration of the asymmetric carbons: C3 (R), C5 (R), C20 (S); Table S1. Crystal data and structure refinement for compounds PP26 (CCDC 1998738).
