# Supplementary material for: New Donor-Acceptor Stenhouse Adducts as Visible and Near Infrared Light Polymerization Photoinitiators
Source: Molecules. 2020 May 15;25(10):2317. doi: 10.3390/molecules25102317 (PMC7287840; doi:10.3390/molecules25102317)
Supplement: Supplementary file 1 [file molecules-25-02317-s001.pdf]

molecules

Supporting Information

New Donor-Acceptor Stenhouse Adducts as Visible and Near Infrared Light Polymerization Photoinitiators

Guillaume Noirbent [1], Yangyang Xu [2,3], Aude-Héloise Bonardi [2,3], Sylvain Duval [4], Didier Gigmes [1], Jacques Lalevée [2,3,*] and Frédéric Dumur [1,*]

[1] Aix Marseille Univ, CNRS, ICR UMR 7273, F-13397 Marseille, France ; guillaume.noirbent@univ-amu.fr; didier.gigmes@univ-amu.fr, frederic.dumur@univ-amu.fr
[2] Université de Haute-Alsace, CNRS, IS2M UMR 7361, F-68100 Mulhouse, France ; ahutxyy@163.com; aude-heloise.bonardi@uha.fr, jacques.lalevee@uha.fr
[3] Université de Strasbourg, 67000 Strasbourg, France
[4] Université de Lille, CNRS, Centrale Lille, Univ. Artois, UMR 8181–UCCS–Unité de Catalyse et Chimie du solide, F-59000 Lille, France; sylvain.duval@univ-lilles.fr
* Correspondence: Jacques.lalevee@uha.fr; frederic.dumur@univ-amu.fr

1. Chemistry

1.1. General Information

All reagents and solvents were purchased from Sigma Aldrich (St. Louis, MO, USA) or Alfa Aesar (Kandel, Germany) and used as received ouri, without further purification. Mass spectroscopy was performed by the Spectropole, analysis laboratory of Aix-Marseille University. ESI mass spectral analyses were recorded with a 3200 QTRAP mass spectrometer (Applied Biosystems SCIEX, Foster City, CA, USA). The HRMS mass spectral analysis was performed with a QStar Elite (Applied Biosystems SCIEX, Foster City, CA, USA) mass spectrometer. Elemental analyses were recorded with an EA 1112 elemental analysis apparatus (Thermo Finnigan, Waltham, MA USA) driven by the Eager 300 software (2.3 version). ^{1}H (400 MHz) and ^{13}C-NMR spectra (100 MHz) were determined at room temperature in 5 mm o.d. tubes on a Avance 400 spectrometer (Bruker, Billarica, MA, USA) of the Spectropole. The ^{1}H and ^{13}C-NMR chemical were referenced to the $CDCl_3$ solvent peaks (7.26 ppm and 77 ppm, respectively). All dyes reported in this work were obtained as solids.

1.2. Synthesis of the dyes

Synthesis of 5-(furan-2-ylmethylene)-2,2-dimethyl-1,3-dioxane-4,6-dione (**PP1**)

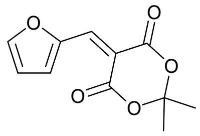

Chemical Formula: $C_{11}H_{10}O_5$
Exact Mass: 222.0528
Molecular Weight: 222.1960

Meldrum's acid (2,2-dimethyl-1,3-dioxane-4,6-dione, 6.41 g, 44.47 mmol, M = 144.13 g/mol) and furfural (4.27 g, 3.68 mL, 44.47 mmol, M = 96.08 g/mol, d = 1.16) were added sequentially to H_2O (200 mL). The heterogeneous mixture was heated to 75 °C and stirred at that temperature for 2 h. The mixture was cooled to room temperature. The precipitated solid was collected by vacuum filtration and washed with cold water. The solid was dissolved in dichloromethane and washed with water.

The organic layer was dried over $MgSO_4$, filtered and the solvent removed to give the title compound (8.40 g, 85% yield, yellow solid). ^{1}H-NMR ($CDCl_3$) δ: 1.76 (s, 6H), 6.73–6.75 (m, 1H), 7.84 (d, 1H, *J* = 1.4 Hz), 8.35 (s, 1H), 8.45 (d, 1H, *J* = 3.8 Hz); ^{13}C-NMR ($CDCl_3$) δ: 27.6, 104.5, 107.7, 115.2, 141.2, 150.2, 150.3, 160.2, 163.2; HRMS (ESI MS) *m*/*z*: theor: 222.0528 found: 222.0530 ($[M]^+$ detected)

Synthesis of 2,2-dimethyl-5-(thiophen-2-ylmethylene)-1,3-dioxane-4,6-dione (**PP2**)

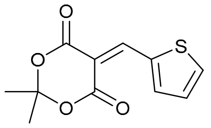

Chemical Formula: $C_{11}H_{10}O_4S$
Exact Mass: 238.0300
Molecular Weight: 238.2570

Meldrum's acid (12.85 g, 89.16 mmol, M = 144.13 g/mol) and 2-thiophenecarboxaldehyde (10 g, 89.16 mmol, M = 112.15 g/mol) were dissolved in ethanol (100 mL) and a few drops of pyridine were added. The reaction was refluxed overnight. After cooling, it was filtered off, washed several times with ethanol and pentane, and dried under vacuum (19.54 g, 92% yield, yellow solid). ^{1}H-NMR ($CDCl_3$) δ: 1.77 (s, 6H), 7.26 (dd, 1H, *J* = 5.1 Hz, *J* = 3.9 Hz), 7.90–7.92 (m, 1H), 7.99–8.02 (m, 1H), 8.66 (d, 1H, *J* = 0.6 Hz); ^{13}C-NMR ($CDCl_3$) δ: 27.6, 104.6, 107.2, 128.3, 136.5, 141.7, 144.8, 149.1, 161.0, 163.5; HRMS (ESI MS) *m*/*z*: theor: 238.0300 found: 238.0304 ($[M]^+$ detected)

Synthesis of 5-((1*H*-pyrrol-2-yl)methylene)-2,2-dimethyl-1,3-dioxane-4,6-dione (**PP3**)

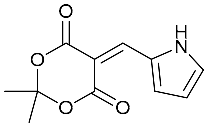

Chemical Formula: $C_{11}H_{11}NO_4$
Exact Mass: 221.0688
Molecular Weight: 221.2120

Meldrum's acid (6.41 g, 44.47 mmol, M = 144.13 g/mol) and 1*H*-pyrrole-2-carbaldehyde (4.22 g, 44.47 mmol, M = 95.10 g/mol) were dissolved in ethanol (50 mL) and a few drops of pyridine were added. The solution was heated at reflux overnight, providing a precipitate. After cooling, it was filtered off, washed several times with ethanol and pentane, and dried under vacuum (8.75 g, 89% yield, yellow solid). ^{1}H-NMR ($CDCl_3$) δ: 1.76 (s, 6H), 6.52–6.54 (m, 1H), 7.10–7.12 (m, 1H), 7.42-7.44 (m, 1H), 8.27 (s, 1H), 12.70 (brs, 1H, NH); ^{13}C-NMR ($CDCl_3$) δ: 27.2, 100.4, 104.3, 114.5, 128.8, 130.2, 131.9, 143.4, 164.2, 164.4; HRMS (ESI MS) *m*/*z*: theor: 221.0688 found: 221.0690 ($[M]^+$ detected)

Synthesis of 2-(furan-2-ylmethylene)-1*H*-indene-1,3(2*H*)-dione (**P4**)

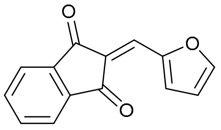

Chemical Formula: $C_{14}H_8O_3$
Exact Mass: 224,0473
Molecular Weight: 224,2150

Indane-1,3-dione (6.5 g, 44.47 mmol, M = 146.15 g/mol) and furfural (4.27 g, 3.68 mL, 44.47 mmol, M = 96.08 g/mol, d = 1.16) were added sequentially to H_2O (200 mL). The heterogeneous mixture was stirred at room temperature for 2 h. The precipitated solid was collected by vacuum filtration and washed with cold water. The solid was dissolved in dichloromethane and washed with water. The organic layer was dried over $MgSO_4$, filtered and the solvent removed (7.18 g, 72% yield, yellow

solid). ^{1}H-NMR ($CDCl_3$) δ 8.57 (d, *J* = 3.7 Hz, 1H), 8.00–7.92 (m, 2H), 7.79–7.76 (m, 3H), 7.74 (s, 1H), 6.71 (ddd, *J* = 3.7, 1.5, 0.6 Hz, 1H).; ^{13}C-NMR ($CDCl_3$) δ 190.0, 189.0, 151.4, 149.0, 142.3, 140.4, 135.1, 134.9, 129.2, 124.8, 124.8, 123.1, 122.9, 114.6; HRMS (ESI MS) *m*/*z*: theor: 224.0473 found: 224.0469 ($[M]^+$ detected)

Synthesis of 2-(thiophen-2-ylmethylene)-1*H*-indene-1,3(2H)-dione (**PP4**)

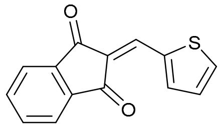

Chemical Formula: $C_{14}H_8O_2S$
Exact Mass: 240.0245
Molecular Weight: 240.2760

Indane-1,3-dione (6.5 g, 44.47 mmol, M = 146.15 g/mol) and 2-thiophenecarboxaldehyde (5 g, 10.30 mmol, M = 112.15 g/mol) were dissolved in ethanol (50 mL) and a few drops of pyridine were added. The solution was heated at reflux overnight, providing a precipitate. After cooling, it was filtered off, washed several times with ethanol and pentane, and dried under vacuum (8.97 g, 84% yield, yellow solid). ^{1}H-NMR (DMSO-d_6) δ: 7.35–7.38 (m, 1H), 7.91–7.99 (m, 4H), 8.11 (s, 1H), 8.24–8.29 (m, 2H); ^{13}C-NMR (DMSO-d_6) δ: 122.7, 122.8, 124.1, 128.9, 135.5, 135.6, 135.8, 136.7, 139.8, 141.4, 143.4, 188.8, 189.4; HRMS (ESI MS) *m*/*z*: theor: 240.0245 found: 240.0243 ($[M]^+$ detected)

Synthesis of 2-((1*H*-pyrrol-2-yl)methylene)-1*H*-indene-1,3(2*H*)-dione (**PP6**)

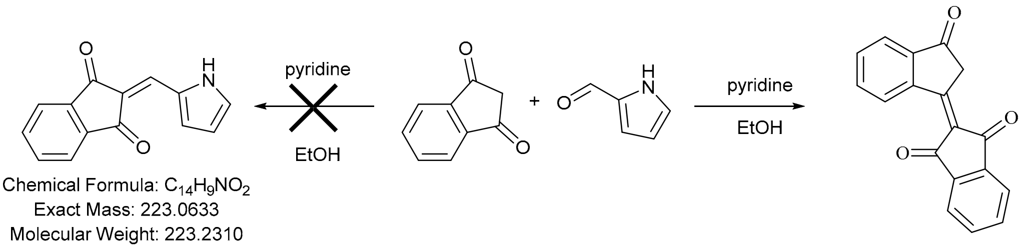

Chemical Formula: $C_{14}H_9NO_2$
Exact Mass: 223.0633
Molecular Weight: 223.2310

Chemical Formula: $C_{18}H_{10}O_3$
Exact Mass: 274.0630
Molecular Weight: 274.2750

Indane-1,3-dione (6.50 g, 44.47 mmol, M = 146.14 g/mol) and 1*H*-pyrrole-2-carbaldehyde (4.22 g, 44.47 mmol, M = 95.10 g/mol) were dissolved in ethanol (50 mL) and a few drops of pyridine were added. The solution was heated at reflux overnight, providing a precipitate. After cooling, it was filtered off, washed several times with ethanol and pentane, and dried under vacuum. The reaction product was identified as bindone, the product resulting from the self-condensation of indane-1,3-dione (5.43 g, 89% yield, grey solid). ^{1}H-NMR ($CDCl_3$) δ: 4.17 (s, 2H), 7.73-7.77 (m, 1H), 7.81–7.88 (m, 3H), 7.94–7.98 (m, 2H), 8.01–8.03 (m, 1H), 9.68 (m, 1H, *J* = 8.1 Hz); ^{13}C-NMR ($CDCl_3$) δ: 43.4, 123.1, 123.4, 123.5, 125.9, 131.7, 134.2, 135.3, 135.3, 135.4, 140.5, 141.3, 141.7, 145.9, 155.4, 189.4, 191.1, 200.9; HRMS (ESI MS) *m*/*z*: theor: 274.0631 found: 274.0633 ($[M]^+$ detected)

Synthesis of 2-(2-(furan-2-ylmethylene)-3-oxo-2,3-dihydro-1*H*-inden-1-ylidene)malononitrile (**PP7**)

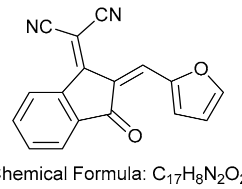

Chemical Formula: $C_{17}H_8N_2O_2$
Exact Mass: 272.0586
Molecular Weight: 272.2630

2-(3-Oxo-2,3-dihydro-1*H*-inden-1-ylidene)malononitrile (2 g, 10.30 mmol, M = 194.19 g/mol) and furfural (0.99 g, 0.85 mL, 10.30 mmol, M = 96.08 g/mol, d = 1.16) were dissolved in ethanol (50 mL) and the solution was heated at 80 °C for 5 min. An orange precipitate formed. After cooling, it was filtered off, washed several times with ethanol, then pentane and dried under vacuum (2.33 g, 83% yield, orange solid). ^{1}H-NMR ($CDCl_3$) δ: 6.77–6.79 (m, 1H), 7.74-7.82 (m, 2H), 7.84 (d, 1H, *J* = 1.2 Hz), 7.93–7.95 (m, 1H), 8.57 (s, 1H), 8.70–8.72 (m, 2H); ^{13}C-NMR ($CDCl_3$) δ: 71.3, 113.8, 114.2, 115.4, 124.0, 124.5, 125.4, 127.3, 130.4, 134.8, 135.3, 137.3, 139.8, 150.2, 150.9, 160.6, 187.1; HRMS (ESI MS) *m*/*z*: theor: 272.0586 found: 272.0585 ($[M]^+$ detected)

Synthesis of 2-(3-oxo-2-(thiophen-2-ylmethylene)-2,3-dihydro-1*H*-inden-1-ylidene)malononitrile (**PP8**)

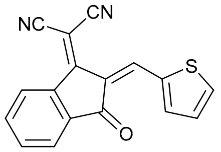

Chemical Formula: $C_{17}H_8N_2OS$
Exact Mass: 288.0357
Molecular Weight: 288.3240

2-(3-Oxo-2,3-dihydro-1*H*-inden-1-ylidene)malononitrile (8.63 g, 44.47 mmol, M = 194.19 g/mol) and 2-thiophenecarboxaldehyde (5 g, 44.47 mmol, M = 112.15 g/mol) were dissolved in ethanol (50 mL) and a few drops of pyridine were added. The solution was heated at reflux overnight, providing a precipitate. After cooling, it was filtered off, washed several times with ethanol and pentane, and dried under vacuum (11.67 g, 91% yield, orange solid). ^{1}H-NMR ($CDCl_3$) δ: 7.30 (dd, 1H, *J* = 4.0 Hz, *J* = 5.0 Hz), 7.77–7.85 (m, 2H), 7.94-8.01 (m, 3H), 8.73-8.75 (m, 1H), 8.96 (s, 1H); ^{13}C-NMR ($CDCl_3$) δ: 71.0, 114.0, 114.2, 123.9, 124.1, 125.5, 128.6, 134.8, 135.4, 137.0, 137.1, 138.3, 139.9, 141.2, 144.3, 160.6, 187.9; HRMS (ESI MS) *m*/*z*: theor: 288.0357 found: 288.0361 ($[M]^+$ detected)

Synthesis of 2-(2-((1H-pyrrol-2-yl)methylene)-3-oxo-2,3-dihydro-1*H*-inden-1-ylidene) malononitrile (**PP9**)

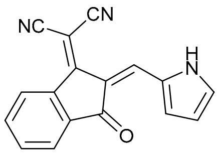

Chemical Formula: $C_{17}H_9N_3O$
Exact Mass: 271.0746
Molecular Weight: 271.2790

2-(3-oxo-2,3-dihydro-1*H*-inden-1-ylidene)malononitrile (8.63 g, 44.47 mmol, M = 194.19 g/mol) and 2-pyrrolecarboxaldehyde (4.23 g, 44.47 mmol, M = 95.10 g/mol) were dissolved in ethanol (50 mL) and a few drops of pyridine were added. The solution was heated at reflux overnight, providing a precipitate. After cooling, it was filtered off, washed several times with ethanol and pentane, and dried under vacuum (10.62 g, 88% yield, orange solid). ^{1}H-NMR (DMSO-d_6) δ: 6.66-6.69 (m, 1H), 7.23–7.25 (m, 1H), 7.84–7.92 (m, 2H), 8.57 (s, 1H), 8.64 (d, 1H, *J* = 1.2 Hz), 8.70-8.72 (m, 2H), 13.35 (brs, 1H, NH); ^{13}C-NMR (CD_2Cl_2) δ: 68.2, 114.6, 114.8, 115.1, 118.8, 123.5, 124.9, 129.1, 130.7, 132.4, 132.8, 134.0, 135.0, 136.5, 139.7, 161.2, 190.1; HRMS (ESI MS) *m*/*z*: theor: 271.0746 found: 271.0748 ($[M]^+$ detected)

Synthesis of 2,2'-(2-(furan-2-ylmethylene)-1*H*-indene-1,3(2*H*)-diylidene)dimalononitrile (**PP10**)

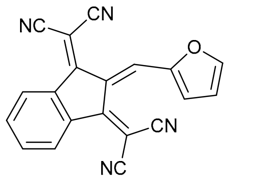

Chemical Formula: $C_{20}H_8N_4O$
Exact Mass: 320.0698
Molecular Weight: 320.3110

2,2'-(1*H*-Indene-1,3(2*H*)-diylidene)dimalononitrile (2 g, 8.25 mmol, M = 242.24 g/mol) and furfural (0.79 g, 0.68 mL, 8.25 mmol, M = 96.08 g/mol, d = 1.16) were dissolved in acetic anhydride (50 mL) and the solution was heated at 80 °C for 2 h. An orange precipitate formed. After cooling, it was filtered off, washed several times with ether, then pentane and dried under vacuum (2.19 g, 83% yield, orange solid). ^{1}H-NMR ($CDCl_3$) δ: 6.71–6.73 (m, 1H), 7.23 (d, 1H, *J* = 3.6 Hz), 7.78–7.84 (m, 3H), 8.21 (s, 1H), 8.51 (dd, 1H, *J* = 8.0 Hz, *J* = 1.4 Hz), 8.63 (d, 1H, *J* = 7.2 Hz); ^{13}C-NMR ($CDCl_3$) δ: 71.8, 78.7, 112.0, 113.0, 113.1, 115.2, 125.2, 126.0, 126.5, 127.2, 127.7, 134.4, 135.1, 136.5, 138.1, 148.4, 149.5, 160.5, 161.5; HRMS (ESI MS) *m*/*z*: theor: 320.0698 found: 320.0700 ($[M]^+$ detected)

Synthesis of 2,2'-(2-(thiophen-2-ylmethylene)-1*H*-indene-1,3(2*H*)-diylidene)dimalononitrile (**PP11**)

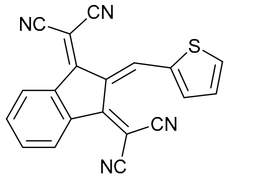

Chemical Formula: $C_{20}H_8N_4S$
Exact Mass: 336.0470
Molecular Weight: 336.3720

2,2'-(1*H*-Indene-1,3(2*H*)-diylidene)dimalononitrile (2 g, 8.25 mmol, M = 242.24 g/mol) and 2-thiophenecarboxaldehyde (0.93 g, 8.25 mmol, M = 112.15 g/mol) were dissolved in acetic anhydride (50 mL) and the solution was heated at 100 °C for 2 h. A red precipitate formed. After cooling, it was filtered off, washed several times with ether, then pentane and dried under vacuum (2.28 g, 82% yield, orange solid). ^{1}H-NMR ($CDCl_3$) δ: 7.23–7.26 (m, 1H), 7.61–7.62 (m, 1H), 7.80–7.87 (m, 3H), 8.52–8.55 (m, 1H), 8.62-8.67 (m, 2H); ^{13}C-NMR ($CDCl_3$) δ: 72.2, 78.4, 112.4, 112.7, 112.8, 113.0, 125.4, 126.2, 129.4, 129.5, 134.9, 135.2, 135.7, 135.9, 136.4, 136.7, 138.0, 138.1160.4, 161.2; HRMS (ESI MS) *m*/*z*: theor: 336.0470 found: 336.0471 ($[M]^+$ detected)

Synthesis of 2,2'-(2-((1*H*-pyrrol-2-yl)methylene)-1*H*-indene-1,3(2*H*)-diylidene)dimalononitrile (**PP12**)

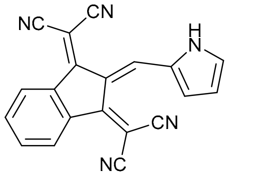

Chemical Formula: $C_{20}H_9N_5$
Exact Mass: 319.0858
Molecular Weight: 319.3270

2,2'-(1*H*-Indene-1,3(2*H*)-diylidene)dimalononitrile (2 g, 8.25 mmol, M = 242.24 g/mol) and 2-pyrrole-carboxaldehyde (0.78 g, 8.25 mmol, M = 95.10 g/mol) were dissolved in acetic anhydride (50 mL) and the solution was heated at 100 °C for 2 h. A red precipitate formed. After cooling, it was filtered off, washed several times with ether, then pentane and finally dried under vacuum (2.32 g, 88% yield).

^{1}H-NMR (DMSO-d6) δ: 6.56–6.57 (m, 1H), 7.28–7.29 (m, 1H), 7.70-7.71 (m, 1H), 7.91–7.93 (m, 2H), 8.35 (s, 1H), 8.41–8.43 (m, 2H); ^{13}C-NMR (DMSO-d6) δ: 69.6, 114.0, 114.6, 115.2, 121.1, 124.6, 129.1, 132.1, 134.1, 137.2, 161.5, 167.0; HRMS (ESI MS) *m*/*z*: theor: 319.0858 found: 319.0856 ([M]+ detected)

Synthesis of 5-(furan-2-ylmethylene)-1,3-dimethylpyrimidine-2,4,6(1*H*,3*H*,5*H*)-trione (**PP13**)

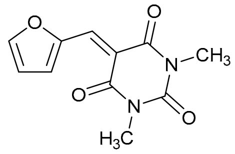

Chemical Formula: $C_{11}H_{10}N_2O_4$
Exact Mass: 234.0641
Molecular Weight: 234.2110

1,3-Dimethylpyrimidine-2,4,6(1*H*,3*H*,5*H*)-trione (6.94 g, 44.47 mmol, M = 156.14 g/mol) furfural (4.27 g, 3.68 mL, 44.47 mmol, M = 96.08 g/mol, d = 1.16) were added sequentially to 200 mL H_2O. The heterogeneous mixture was stirred at room temperature for 2 h. The precipitated solid was collected by vacuum filtration and washed with cold water. The solid was dissolved in dichloromethane and washed with water. The organic layer was dried over $MgSO_4$, filtered and the solvent removed (9.27 g, 89% yield, orange solid). ^{1}H-NMR ($CDCl_3$) δ: 3.40 (s, 3H), 3.41 (s, 3H), 6.73–6.74 (m, 1H), 7.84–7.85 (d, 1H, *J* = 1.4 Hz), 8.43 (s,1H), 8.64 (d, 1H, *J* = 3.8 Hz); ^{13}C-NMR ($CDCl_3$) δ: 28.2, 28.9, 111.5, 115.1, 127.9, 140.9, 150.3, 151.2, 160.8, 162.4; HRMS (ESI MS) *m*/*z*: theor: 234.0641 found: 234.0642 ([M]+ detected)

Synthesis of 1,3-dimethyl-5-(thiophen-2-ylmethylene)pyrimidine-2,4,6(1*H*,3*H*,5*H*)-trione (**PP14**)

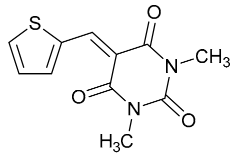

Chemical Formula: $C_{11}H_{10}N_2O_3S$
Exact Mass: 250.0412
Molecular Weight: 250.2720

1,3-Dimethylpyrimidine-2,4,6(1*H*,3*H*,5*H*)-trione (6.94 g, 44.47 mmol, M = 156.14 g/mol) and 2-thiophenecarboxaldehyde (5 g, 44.47 mmol, M = 112.15 g/mol) were dissolved in ethanol (50 mL) and a few drops of pyridine were added. The solution was heated at reflux overnight, providing a precipitate. After cooling, it was filtered off, washed several times with ethanol and pentane, and dried under vacuum (9.13 g, 82% yield, orange solid). ^{1}H-NMR ($CDCl_3$) δ: 3.42 (s, 3H), 3.43 (s, 3H), 7.29 (dd, 1H, *J* = 5.1 Hz, *J* = 3.9 Hz), 7.90–7.91 (m, 1H), 7.99–8.02 (m, 1H), 8.75 (d, 1H, *J* = 0.7 Hz); ^{13}C-NMR ($CDCl_3$) δ: 28.1, 28.9, 110.6, 128.2, 137.0, 141.8, 145.4, 149.0, 151.3, 161.7, 162.6; HRMS (ESI MS) *m*/*z*: theor: 250.0412 found: 250.0411 ([M]+ detected)

Synthesis of 5-((1H-pyrrol-2-yl)methylene)-1,3-dimethylpyrimidine-2,4,6(1*H*,3*H*,5*H*)-trione (**PP15**)

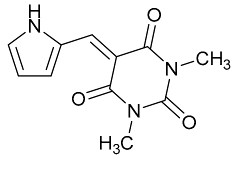

Chemical Formula: $C_{11}H_{11}N_3O_3$
Exact Mass: 233.0800
Molecular Weight: 233.2270

1,3-Dimethylpyrimidine-2,4,6(1*H*,3*H*,5*H*)-trione (6.94 g, 44.47 mmol, M = 156.14 g/mol) and 1*H*-pyrrole-2-carbaldehyde (4.22 g, 44.47 mmol, M = 95.10 g/mol) were dissolved in ethanol (50 mL) and a few drops of pyridine were added. The solution was heated at reflux overnight, providing a precipitate. After cooling, it was filtered off, washed several times with ethanol and pentane, and dried under vacuum (9.33 g, 90% yield, orange solid). ^{1}H-NMR ($CDCl_3$) δ: 3.40 (s, 3H), 3.42 (s, 3H), 6.53–6.55 (m, 1H), 7.13-7.15 (m, 1H), 7.40–7.41 (m, 1H), 13.28 (brs, 1H, NH); ^{13}C-NMR ($CDCl_3$) δ: 28.3, 28.8, 105.6, 114.6, 129.5, 130.0, 131.3, 142.9, 151.5, 163.3, 163.7; HRMS (ESI MS) *m*/*z*: theor: 233.0800 found: 233.0798 ($[M]^+$ detected)

Synthesis of 1,3-diethyl-5-(furan-2-ylmethylene)-2-thioxodihydropyrimidine-4,6(1*H*,5*H*)-dione (**PP16**)

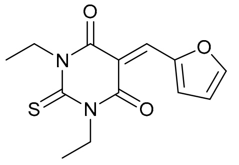

Chemical Formula: $C_{13}H_{14}N_2O_3S$
Exact Mass: 278,0725
Molecular Weight: 278,3260

1,3-Diethyl-2-thiobarbituric acid (10.00 g, 49.94 mmol, M = 200.26 g/mol) and furfural (4.8 g, 4.14 mL, 49.94 mmol, M = 96.08 g/mol, d = 1.16) were added sequentially to 200 mL H2O. The heterogeneous mixture was stirred at room temperature for 2 h. The precipitated solid was collected by vacuum filtration and washed with cold water. The solid was dissolved in dichloromethane and washed with water. The organic layer was dried over $MgSO_4$, filtered and the solvent removed (13.5 g, 97% yield, orange solid). ^{1}H-NMR ($CDCl_3$) δ 8.69 (d, *J* = 3.9 Hz, 1H), 8.42 (s, 1H), 7.88 (d, *J* = 1.3 Hz, 1H), 6.75 (ddd, *J* = 3.8, 1.6, 0.7 Hz, 1H), 4.56 (qd, *J* = 7.0, 2.9 Hz, 4H), 1.31 (td, *J* = 7.0, 4.0 Hz, 6H).; ^{13}C-NMR ($CDCl_3$) δ 178.7, 160.7, 158.7, 151.6, 150.9, 141.8, 128.7, 115.4, 112.3, 44.0, 43.3, 12.4, 12.3; HRMS (ESI MS) *m*/*z*: theor: 278.0725 found: 278.0729 ($[M]^+$ detected)

Synthesis of 1,3-diethyl-5-(thiophen-2-ylmethylene)-2-thioxodihydropyrimidine-4,6(1*H*,5*H*)-dione (**PP17**)

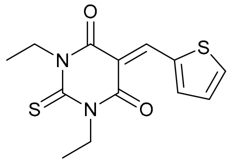

Chemical Formula: $C_{13}H_{14}N_2O_2S_2$
Exact Mass: 294.0497
Molecular Weight: 294.3870

1,3-Diethyl-2-thiobarbituric acid (2. g, 9.99 mmol, M = 200.26 g/mol) and 2-thiophenecarboxaldehyde (1.12 g, 9.99 mmol, M = 112.15 g/mol) were dissolved in ethanol (50 mL) and a few drops of pyridine were added. The solution was heated at 100 °C for 2 h. A yellow precipitate formed. After cooling, it was filtered off, washed several times with ether, then pentane and dried under vacuum (2.53 g, 86% yield, orange solid). ^{1}H-NMR ($CDCl_3$) δ: 1.32 (t, 3H, *J* = 7.1 Hz), 1.34 (t, 3H, *J* = 7.1 Hz), 4.58 (q, 2H, *J* = 6.9 Hz), 4.60 (q, 2H, *J* = 6.9 Hz), 7.31 (dd, 1H, *J* = 5.1 Hz, *J* = 3.9 Hz), 7.92–7.94 (m, 1H), 8.02–8.05 (m, 1H), 8.76 (d, 1H, *J* = 0.6 Hz); ^{13}C-NMR ($CDCl_3$) δ: 12.36, 12.45, 43.2, 44.0, 111.5, 128.5, 137.5, 142.5, 145.6, 150.1, 159.7, 160.9, 178.8; HRMS (ESI MS) *m*/*z*: theor: 294.0497 found: 294.0495 ($[M]^+$ detected)

Synthesis of 5-((1*H*-pyrrol-2-yl)methylene)-1,3-diethyl-2-thioxodihydropyrimidine-4,6(1*H*,5*H*)-dione (**PP18**)

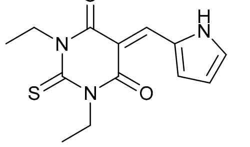

Chemical Formula: $C_{13}H_{15}N_3O_2S$
Exact Mass: 277.0885
Molecular Weight: 277.3420

1,3-Diethyl-2-thiobarbituric acid (2. g, 9.99 mmol, M = 200.26 g/mol) and 2-furanecarboxaldehyde (0.95 g, 9.99 mmol, M = 95.10 g/mol) were dissolved in ethanol (50 mL) and a few drops of pyridine were added. The solution was heated at 100 °C for 2 h. A yellow precipitate formed. After cooling, it was filtered off, washed several times with ether, then pentane and dried under vacuum (2.35 g, 85% yield, orange solid). ^{1}H-NMR (DMSO-d$_6$) δ: 1.21 (t, 3H, *J* = 6.9 Hz), 1.25 (t, 3H, *J* = 6.9 Hz), 4.45 (q, 2H, *J* = 7.0 Hz), 4.52 (q, 2H, *J* = 7.0 Hz), 6.62–6.63 (m, 1H), 7.79 (s, 1H), 8.26 (s, 1H), 13.0 (brs, 1H, NH); ^{13}C-NMR (DMSO-d$_6$) δ: 12.1, 12.2, 42.9, 43.2, 105.9, 115.1, 129.6, 135.3, 142.6, 161.0, 178.2; HRMS (ESI MS) *m*/*z*: theor: 277.0885 found: 277.0887 ([M]$^+$ detected).

Synthesis of 5-(3-(furan-2-yl)allylidene)-2,2-dimethyl-1,3-dioxane-4,6-dione (**PP19**)

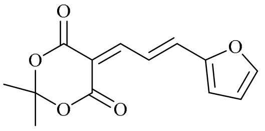

Chemical Formula: $C_{13}H_{12}O_5$
Exact Mass: 248.0685
Molecular Weight: 248.2340

2,2-Dimethyl-1,3-dioxane-4,6-dione (1.19 g, 8.25 mmol, M = 144.13 g/mol) and 3-(2-furyl)acrolein (1.01 g, 8.25 mmol, M = 122.12 g/mol) were dissolved in ethanol (50 mL) and a few drops of pyridine were added. The solution was heated at 80 °C overnight. A yellow precipitate formed. After cooling, it was filtered off, washed several times with ether, then pentane and dried under vacuum (1.78 g, 87% yield,). ^{1}H-NMR (CDCl$_3$) δ: 1.74 (s, 6H), 6.54–6.56 (m, 1H), 6.85 (d, 1H, *J* = 3.5 Hz), 7.08–7.20 (m, 1H), 7.61–7.62 (m, 1H), 8.05-8.17 (m, 2H); ^{13}C-NMR (CDCl$_3$) δ: 27.6, 104.6, 110.8, 113.4, 118.2, 122.8, 138.6, 147.0, 151.9, 157.0, 160.7, 163.0; HRMS (ESI MS) *m*/*z*: theor: 248.0685 found: 248.0686 ([M]$^+$ detected)

Synthesis of 2-(3-(furan-2-yl)allylidene)-1*H*-indene-1,3(2*H*)-dione (**PP20**)

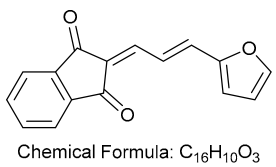

Chemical Formula: $C_{16}H_{10}O_3$
Exact Mass: 250.0630
Molecular Weight: 250.2530

1,3-Indanedione (1.51 g, 10.30 mmol, M = 146.14 g/mol) and 3-(2-furyl)acrolein (1.26 g, 10.30 mmol, M = 122.12 g/mol) were dissolved in ethanol (50 mL) and no base was added. The solution was heated at 80 °C for 30 min. A brown precipitate formed. After cooling, it was filtered off, washed several times with ether, then pentane and dried under vacuum (2.24 g, 87% yield, red solid). ^{1}H-NMR (CDCl$_3$) δ: 6.53–6.54 (m, 1H), 6.77 (d, 1H, *J* = 3.4 Hz), 7.08 (d, 1H, *J* = 15.3 Hz), 7.56 (d, 1H, *J* = 12.4 Hz), 7.58–7.59 (m, 1H), 7.75–7.78 (m, 2H), 7.94–7.96 (m, 2H), 8.25 (dd, 1H, *J* = 15.3 Hz, *J* = 12.4 Hz); ^{13}C-NMR

($CDCl_3$) δ: 113.1, 116.1, 122.1, 122.9, 123.0, 127.7, 134.8, 135.0, 136.0, 140.9, 142.2, 143.9, 146.0, 152.4; HRMS (ESI MS) *m*/*z*: theor: 250.0630 found: 250.0634 ($[M]^+$ detected)

Synthesis of 2-((*Z*)-2-((*E*)-3-(furan-2-yl)allylidene)-3-oxo-2,3-dihydro-1*H*-inden-1-ylidene) malononitrile (**PP21**)

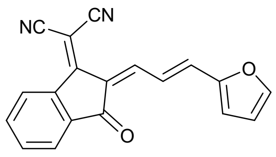

Chemical Formula: $C_{19}H_{10}N_2O_2$
Exact Mass: 298.0742
Molecular Weight: 298.3010

2-(3-Oxo-2,3-dihydro-1*H*-inden-1-ylidene)malononitrile (2 g, 10.30 mmol, M = 194.19 g/mol) and 3-(2-furyl)acrolein (1.26 g, 10.30 mmol, M = 122.12 g/mol) were dissolved in ethanol (50 mL) and a few drops of pyridine were added. The solution was heated at 80 °C for 30 min. A brown precipitate formed. After cooling, it was filtered off, washed several times with ether, then pentane and dried under vacuum (2.58 g, 84% yield, red solid). ^{1}H-NMR ($CDCl_3$) δ: 6.58 (s, 1H), 6.86 (s, 1H), 7.16 (d, 1H, *J* = 14.9 Hz), 1.65 (s, 1H), 7.74–7.82 (m, 2H), 7.93 (d, 1H, *J* = 6.0 Hz), 8.42 (d, 1H, *J* = 11.5 Hz), 8.62 (d, 1H, *J* = 13.5 Hz), 8.70 (d, 1H, *J* = 7.1 Hz); ^{13}C-NMR ($CDCl_3$) δ: 70.7, 113.6, 114.2, 114.4, 118.1, 122.6, 124.0, 125.4, 126.4, 134.6, 135.2, 137.4, 139.9, 146.6, 147.0, 152.5, 159.5, 189.0; ^{1}H-NMR (DMSO-d_6) δ: 6.78–6.79 (m, 1H), 7.16 (d, 1H, *J* = 3.4 Hz), 7.51 (d, 1H, *J* = 15.0 Hz), 7.89–7.92 (m, 2H), 7.97-8.01 (m, 1H), 8.08 (s, 1H), 8.31 (d, 1H, *J* = 11.9 Hz), 8.46 (dd, 1H, *J* = 15.0 Hz, *J* = 11.9 Hz), 8.55 (d, 1H, *J* = 8.0 Hz); HRMS (ESI MS) *m*/*z*: theor: 298.0742 found: 298.0744 ($[M]^+$ detected)

Synthesis of 2,2'-(2-(3-(furan-2-yl)allylidene)-1*H*-indene-1,3(2*H*)-diylidene)dimalononitrile (**PP22**)

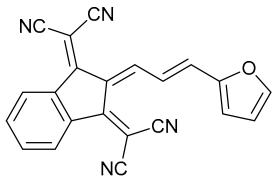

Chemical Formula: $C_{22}H_{10}N_4O$
Exact Mass: 346.0855
Molecular Weight: 346.3490

2,2'-(1*H*-Indene-1,3(2*H*)-diylidene)dimalononitrile (2 g, 8.25 mmol, M = 242.24 g/mol) and 3-(2-furyl)acrolein (1.01 g, 8.25 mmol, M = 122.12 g/mol) were dissolved in acetic anhydride (50 mL) and the solution was heated at 80 °C for 3 h. An orange precipitate formed. After cooling, it was filtered off, washed several times with ether, then pentane and dried under vacuum (2.31 g, 81% yield, red solid). ^{1}H-NMR (DMF-d_7) δ: 6.80–6.82 (m, 1H), 7.28 (d, 1H, *J* = 3.5 Hz), 7.42–7.49 (dd, 1H, *J* = 14.7 Hz, *J* = 12.0 Hz), 7.59 (d, 1H, *J* = 14.7 Hz), 8.01–8.08 (m, 3H), 8.19 (d, 1H, *J* = 12.0 Hz), 8.49–8.55 (m, 2H); Anal. Calc. for $C_{22}H_{10}N_4O$: C, 76.3; H, 2.9; O, 4.6; Found: C, 76.4; H, 2.8; O, 4.7%; HRMS (ESI MS) *m*/*z*: theor: 346.0855 found: 346.0856 ($[M]^+$ detected)

Synthesis of 5-(3-(furan-2-yl)allylidene)-1,3-dimethylpyrimidine-2,4,6(1*H*,3*H*,5*H*)-trione (**PP23**)

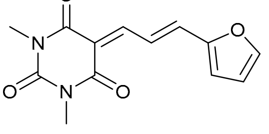

Chemical Formula: $C_{13}H_{12}N_2O_4$
Exact Mass: 260.0797
Molecular Weight: 260.2490

1,3-Dimethylbarbituric acid (1.61 g, 10.30 mmol, M = 156.14 g/mol) and 3-(2-furyl)acrolein (1.26 g, 10.30 mmol, M = 122.12 g/mol) were dissolved in ethanol (50 mL) and no base was added. The solution was heated at 80 °C for 30 min. A brown precipitate formed. After cooling, it was filtered off, washed several times with ether, then pentane and dried under vacuum (2.36 g, 88% yield, red solid). ^{1}H-NMR (DMSO d$_6$) δ: 3.21 (s, 6H), 6.73–6.75 (m, 1H), 7.05 (d, 1H, *J* = 3.5 Hz), 7.59 (d, 1H, *J* = 15.0 Hz), 8.00–8.01 (d, 1H, *J* = 1.4 Hz), 8.07 (d, 1H, *J* = 12.3 Hz), 8.26 (dd, 1H, *J* = 15.0 Hz, *J* = 12.3 Hz); ^{13}C-NMR (DMSO d$_6$) δ: 27.6, 28.2, 113.7, 114.5, 118.4, 122.1, 138.3, 147.6, 151.1, 151.8, 154.1, 161.5, 161.8; HRMS (ESI MS) *m*/*z*: theor: 260.0797 found: 260.0799 ([M]$^+$ detected)

Synthesis of 1,3-diethyl-5-(3-(furan-2-yl)allylidene)-2-thioxodihydropyrimidine-4,6(1*H*,5*H*)- dione (**PP24**)

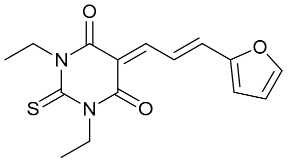

Chemical Formula: $C_{15}H_{16}N_2O_3S$
Exact Mass: 304.0882
Molecular Weight: 304.3640

1,3-Diethyl-2-thiobarbituric acid (2.06 g, 10.30 mmol, M = 200.26 g/mol) and 3-(2-furyl)acrolein (1.26 g, 10.30 mmol, M = 122.12 g/mol) were dissolved in ethanol (50 mL) and no base was added. The solution was heated at 80 °C for 30 min. A brown precipitate formed. After cooling, it was filtered off, washed several times with ether, then pentane and dried under vacuum (2.48 g, 79% yield, red solid). ^{1}H-NMR (CDCl$_3$) δ: 1.30 (t, 3H, *J* = 7.0 Hz), 1.31 (t, 3H, *J* = 7.0 Hz), 4.51–4.59 (m, 4H), 6.56–6.58 (m, 1H), 6.88 (d, 1H, *J* = 3.5 Hz), 7.20 (d, 1H, *J* = 15.1 Hz), 7.62 (d, 1H, *J* = 1.5 Hz), 8.14 (d, 1H, *J* = 12.5 Hz), 8.44 (dd, 1H, *J* = 15.1 Hz, *J* = 12.5 Hz); ^{13}C-NMR (CDCl$_3$) δ: 12.39, 12.44, 43.2, 43.7, 113.6, 114.8, 118.3, 124.0, 139.1, 147.1, 152.4, 157.5, 159.7, 160.6, 178.9; HRMS (ESI MS) *m*/*z*: theor: 304.0882 found: 304.0884 ([M]$^+$ detected)

Synthesis of 2-(4-cyano-3-(diethylamino)-1-(furan-2-yl)-1,2-dihydro-9*H*-indeno[2,1-*c*]pyridine-9-ylidene)malononitrile (**PP25**)

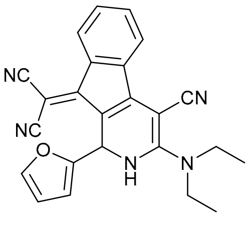

Chemical Formula: $C_{24}H_{19}N_5O$
Exact Mass: 393.1590
Molecular Weight: 393.4500

2-(2-(Furan-2-ylmethylene)-3-oxo-2,3-dihydro-1*H*-inden-1-ylidene)malononitrile (0.59 g, 1.84 mmol, M = 320.31 g/mol) was dissolved in THF (50 mL) and diethylamine (0.14 g, 0.19 mL, 1.84 mmol, M = 73.14 g/mol, d = 0.707) was added. The solution was stirred at room temperature for 10 min. THF was partially evaporated. Addition of ether precipitated a black solid that was filtered off, washed several times with ether and dried under vacuum (644 mg, 89% yield, blue solid). ^{1}H-NMR (CDCl$_3$) δ: 1.33 (t, 6H, *J* = 7.1 Hz), 3.60–3.69 (m, 4H), 6.19 (d, 1H, *J* = 3.2 Hz), 6.30–6.33 (m, 2H), 6.65–6.85 (brs, 1H, NH), 7.32–7.38 (m, 3H), 8.11–8.19 (m, 2H); ^{13}C-NMR (CDCl$_3$) δ: 12.9, 14.6, 45.7, 53.2, 106.3, 113.4, 118.1, 118.2, 122.1, 123.8, 127.3, 132.2, 133.7, 136.6, 141.1, 148.4, 152.8, 159.8, 160.0, 191.7; HRMS (ESI MS) *m*/*z*: theor: 393.1590 found: 393.1594 ([M]$^+$ detected)

Synthesis of 10-(dicyanomethylene)-5-(methyl(phenyl)amino)-8-oxo-6,7,8,10-tetrahydro- 5*H*-4*b*,7-methanobenzo[*a*]azulene-11,11-dicarbonitrile (**PP26**)

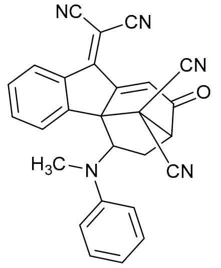

Chemical Formula: $C_{27}H_{17}N_5O$
Exact Mass: 427.1433
Molecular Weight: 427.4670

2-(2-(Furan-2-ylmethylene)-3-oxo-2,3-dihydro-1*H*-inden-1-ylidene)malononitrile (0.5 g, 1.84 mmol, M = 272.26 g/mol) was dissolved in THF (50 mL) and *N*-methylbenzylamine (0.22 g, 0.24 mL, 1.84 mmol, M = 121.18 g/mol, d = 0.939) was added. The solution was stirred at room temperature for 10 min. THF was partially evaporated. Addition of ether precipitated a black solid that was filtered off, washed several times with ether and dried under vacuum (652 mg, 83% yield, blue solid). ^{1}H-NMR ($CDCl_3$) δ: 2.28–2.33 (m, 1H), 2.59 (s, 3H), 2.98–3.06 (m, 1H), 3.70–3.72 (m, 1H), 5.19–5.23 (m, 1H), 6.54 (d, 2H, *J* = 8.8 Hz), 6.81 (t, 1H, *J* = 7.3 Hz), 7.01–7.07 (m, 2H), 7.56 (d, 1H, *J* = 1.3 Hz), 7.61 (td, 1H, *J* = 7.5 Hz, *J* = 1.1 Hz), 7.78 (td, 1H, *J* = 7.5 Hz, *J* = 1.1 Hz), 8.11 (d, 1H, *J* = 7.8 Hz), 8.46 (d, 1H, *J* = 8.1 Hz); ^{1}H-NMR (Acetone–d_6) δ: 2.55–2.60 (m, 1H), 2.75 (s, 3H), 3.22–3.22 (m, 1H), 4.16 (d, 1H, *J* = 8.0 Hz), 5.52–5.56 (m, 1H), 6.68–6.74 (m, 3H), 6.99–7.04 (m, 2H), 7.52 (d, 1H, *J* = 1.4 Hz), 7.74 (td, 1H, *J* = 8.4 Hz, *J* = 1.0 Hz), 7.91 (td, 1H, *J* = 8.4 Hz, *J* = 1.0 Hz), 8.20 (d, 1H, *J* = 7.9 Hz), 8.44 (d, 1H, *J* = 8.2 Hz); ^{1}H-NMR (THF-d_8) δ: 2.41–2.45 (m, 1H), 2.66 (s, 3H), 2.98–3.12 (m, 1H), 3.96 (d, 1H, *J* = 8.0 Hz), 5.39–5.43 (m, 1H), 6.62–6.68 -m, 3H), 6.95–6.99 (m, 2H), 7.50 (d, 1H, *J* = 1.4 Hz), 7.65 (td, 1H, *J* = 8.4 Hz, *J* = 1.0 Hz), 7.79 (td, 1H, *J* = 8.4 Hz, *J* = 1.0 Hz), 8.16 (d, 1H, *J* = 7.9 Hz), 8.44 (d, 1H, *J* = 8.1 Hz); ^{13}C-NMR ($CDCl_3$) δ: 27.3, 37.2, 44.7, 55.2, 63.8, 69.1, 77.3, 110.8, 112.37, 112.39, 112.5, 118.4, 122.2, 124.3, 126.7, 127.1, 129.2, 131.9, 136.7, 137.0, 140.7, 149.5, 155.2, 158.4, 192.0; HRMS (ESI MS) *m*/*z*: theor: 427.1433 found: 427.1435 ($[M]^+$ detected)

Synthesis of 2-((*Z*)-2-((2*Z*,4*E*)-5-(diethylamino)-2-hydroxypenta-2,4-dien-1-ylidene)-3-oxo-2,3-dihydro-1*H*-inden-1-ylidene)malononitrile (**DASA1**)

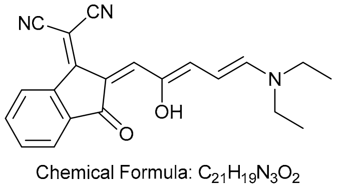

Chemical Formula: $C_{21}H_{19}N_3O_2$
Exact Mass: 345.1477
Molecular Weight: 345.4020

2-(2-(Furan-2-ylmethylene)-3-oxo-2,3-dihydro-1H-inden-1-ylidene)malononitrile (0.5 g, 1.84 mmol, M = 272.26 g/mol) was dissolved in THF (50 mL) and diethylamine (0.14 g, 0.19 mL, M = 73.14 g/mol, d = 0.707) was added. The solution was stirred at room temperature for 10 min. THF was partially evaporated. Addition of ether precipitated a black solid that was filtered off, washed several times with ether and dried under vacuum (540 mg, 85% yield, blue solid). ^{1}H-NMR (THF-d_8) δ: 1.37 (t, 3H, *J* = 7.3 Hz), 1.41 (t, 3H, *J* = 7.3 Hz), 3.71 (q, 2H, *J* = 7.3 Hz), 3.77 (q, 2H, *J* = 7.3 Hz), 6.48 (t, 1H, *J* = 11.9 Hz), 7.07 (dd, 1H, *J* = 12.9 Hz, *J* = 1.4 Hz), 7.45–7.55 (m, 3H), 8.06 (d, 1H, *J* = 11.8 Hz), 8.38 (d, 1H, *J* = 7.4 Hz), 12.29 (s, 1H); ^{13}C-NMR (THF–d_8) δ: 12.9, 14.3, 26.4, 45.7, 53.3, 108.1, 112.7, 118.2, 118.5, 122.0, 123.7, 124.9, 132.1, 133.5, 137.2, 141.7, 149.0, 154.1, 159.1, 162.9, 191.4; HRMS (ESI MS) *m*/*z*: theor: 345.1477 found: 345.1480 ($[M]^+$ detected)

Synthesis of 2-((*Z*)-2-((2*Z*,4*E*)-2-hydroxy-5-(piperidin-1-yl)penta-2,4-dien-1-ylidene)-3-oxo -2,3-dihydro-1*H*-inden-1-ylidene)malononitrile (**DASA2**)

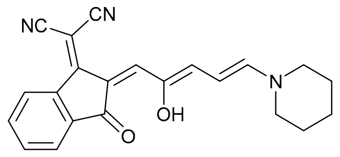

Chemical Formula: $C_{22}H_{19}N_3O_2$
Exact Mass: 357.1477
Molecular Weight: 357.4130

2-(2-(Furan-2-ylmethylene)-3-oxo-2,3-dihydro-1*H*-inden-1-ylidene)malononitrile (0.5 g, 1.84 mmol, M = 272.26 g/mol) was dissolved in THF (50 mL) and piperidine (0.16 g, 0.18 mL, M = 85.15 g/mol, d = 0.861) was added. The solution was stirred at room temperature for 10 min. THF was partially evaporated. Addition of ether precipitated a black solid that was filtered off, washed several times with ether and dried under vacuum (480 mg, 73% yield, blue solid). ^{1}H-NMR (DMSO-d$_6$) δ: 1.56–1.78 (m, 6H), 3.77–3.87 (m, 4H), 6.61 (t, 1H, *J* = 11.3 Hz), 6.81 (s, 1H), 7.12 (d, 1H, *J* = 6.9 Hz), 7.16 (d, 1H, *J* = 13.5 Hz), 7.23–7.38 (m, 3H), 7.39–7.50 (m, 3H), 7.53–7.58 (m, 1H), 7.93 (d, 1H, *J* = 7.2 Hz), 7.97 (d, 1H, *J* = 7.4 Hz), 8.12 (d, 1H, *J* = 7.5 Hz), 8.46 (d, 1H, *J* = 11.2 Hz), 12.16 (s, 1H); ^{13}C-NMR (DMSO–d$_6$) δ: 21.5, 22.2, 43.8, 50.0, 62.8, 102.4, 118.1, 118.7, 119.5, 120.2, 128.9, 129.5, 130.9, 131.0, 134.2, 135.5, 140.2, 141.3, 155.2, 186.5, 189.1; HRMS (ESI MS) *m*/*z*: theor: 357.1477 found: 357.1478 ([M]$^+$ detected)

Synthesis of 2-((*Z*)-2-((2*Z*,4*E*)-5-(*bis*(2-hydroxyethyl)amino)-2-hydroxypenta-2,4-dien-1-ylidene)- 3-oxo-2,3-dihydro-1*H*-inden-1-ylidene)malononitrile (**DASA3**)

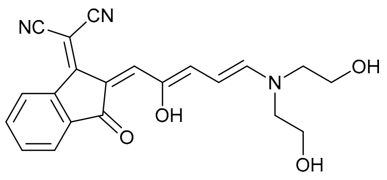

Chemical Formula: $C_{21}H_{19}N_3O_4$
Exact Mass: 377.1376
Molecular Weight: 377.4000

2-(2-(Furan-2-ylmethylene)-3-oxo-2,3-dihydro-1H-inden-1-ylidene)malononitrile (0.5 g, 1.84 mmol, M = 272.26 g/mol) was dissolved in THF (50 mL) and diethanolamine (0.19 g, 0.18 mL, M = 105.14 g/mol, d = 1.097) was added. The solution was stirred at room temperature for 10 min. THF was partially evaporated. Addition of ether precipitated a black solid that was filtered off, washed several times with ether and dried under vacuum (541 mg, 78% yield, blue solid). ^{1}H-NMR (DMSO-d$_6$) δ: 3.71–3.76 (m, 4H), 3.82 (t, 4H, *J* = 5.2 Hz), 5.13 (t, 2H, *J* = 4.8 Hz, OH), 6.58 (t, 1H, *J* = 11.3 Hz), 6.82 (s, 1H), 7.20 (d, 1H, *J* = 13.5 Hz), 7.45–7.50 (m, 2H), 7.53–7.58 (m, 1H), 8.12 (d, 1H, *J* = 7.5 Hz), 8.45 (d, 1H, *J* = 11.3 Hz), 12.1 (s, 1H); ^{13}C-NMR (DMSO–d$_6$) δ: 57.9, 58.2, 60.6, 110.2, 110.7, 117.7, 118.1, 118.2, 120.9, 121.9, 131.4, 132.9, 135.1, 139.8, 146.7, 153.6, 157.4, 167.2, 189.9; HRMS (ESI MS) *m*/*z*: theor: 377.1376 found: 377.1373 ([M]$^+$ detected).

Synthesis of 2-((*Z*)-2-((2*Z*,4*E*)-5-(dibenzylamino)-2-hydroxypenta-2,4-dien-1-ylidene)-3-oxo -2,3-dihydro-1*H*-inden-1-ylidene)malononitrile (**DASA4**)

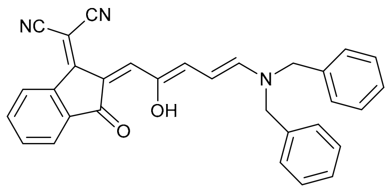

Chemical Formula: $C_{31}H_{23}N_3O_2$
Exact Mass: 469.1790
Molecular Weight: 469.5440

2-(2-(Furan-2-ylmethylene)-3-oxo-2,3-dihydro-1H-inden-1-ylidene)malononitrile (0.5 g, 1.84 mmol, M = 272.26 g/mol) was dissolved in THF (50 mL) and dibenzylamine (0.36 g, 0.35 mL, M = 197.28 g/mol, d = 1.026) was added. The solution was stirred at room temperature for 10 min. THF was partially evaporated. Addition of ether precipitated a black solid that was filtered off, washed several times with ether and dried under vacuum (708 mg, 78% yield, blue solid). ^{1}H-NMR ($CDCl_3$) δ: 4.74 (s, 2H), 4.78 (s, 2H), 6.62 (t, 1H, *J* = 12.2 Hz), 7.10 (dd, 1H, *J* = 12.6 Hz, *J* = 1.5 Hz), 7.28–7.44 (m, 12H), 7.46–7.50 (m, 1H), 7.52–7.57 (m, 2H), 8.32 (d, 1H, *J* = 11.9 Hz), 8.38–8.40 (m, 1H), 12.13 (s, 1H); ^{13}C-NMR ($CDCl_3$) δ: 52.9, 61.3, 107.1, 113.7, 117.8, 118.1, 122.4, 124.0, 127.8, 128.7, 129.2, 129.3, 129.7, 129.9, 130.0, 132.5, 134.0, 135.1, 135.3, 137.1, 141.6, 149.6, 153.8, 159.5, 163.6, 191.7; HRMS (ESI MS) *m*/*z*: theor: 469.1790 found: 469.1786 ($[M]^+$ detected)

Synthesis of 2-((*Z*)-2-((2*Z*,4*E*)-2-hydroxy-5-(methyl(phenyl)amino)penta-2,4-dien-1-ylidene)-3-oxo-2,3-dihydro-1*H*-inden-1-ylidene)malononitrile (**DASA5**)

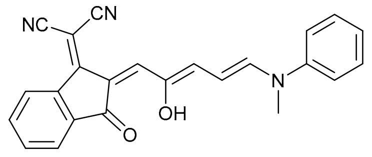

Chemical Formula: $C_{24}H_{17}N_3O_2$
Exact Mass: 379.1321
Molecular Weight: 379.4190

2-(2-(Furan-2-ylmethylene)-3-oxo-2,3-dihydro-1H-inden-1-ylidene)malononitrile (0.5 g, 1.84 mmol, M = 272.26 g/mol) was dissolved in THF (50 mL) and N-methylaniline (0.20 g, 0.20 mL, M = 107.15 g/mol, d = 0.989) was added. The solution was stirred at room temperature for 10 min. THF was partially evaporated. Addition of ether precipitated a black solid that was filtered off, washed several times with ether and dried under vacuum (600 mg, 86% yield, blue solid). ^{1}H-NMR ($CDCl_3$) δ: 3.53 (s, 3H), 6.29 (t, 1H, *J* = 14.4 Hz), 6.75 (d, 1H, *J* = 12.2 Hz), 7.18 (d, 2H, *J* = 6.4 Hz), 7.26 (t, 1H, *J* = 7.2 Hz), 7.41 (t, 2H, *J* = 6.4 Hz), 7.46-7.59 (m, 4H), 7.65 (d, 1H, *J* = 7.0 Hz), 8.42 (d, 1H, *J* = 7.6 Hz), 11.95 (OH); Anal. Calc. for $C_{24}H_{17}N_3O_2$: C, 76.0; H, 4.5; O, 8.4; Found: C, 76.1; H, 4.5; O, 8.5%; HRMS (ESI MS) *m*/*z*: theor: 379.1321 found: 379.1322 ($[M]^+$ detected)

Synthesis of 5-((2*Z*,4*E*)-5-(diethylamino)-2-hydroxypenta-2,4-dien-1-ylidene)-2,2-dimethyl-1,3-dioxane-4,6-dione (**DASA6**)

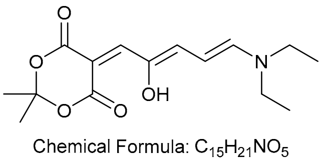

Chemical Formula: $C_{15}H_{21}NO_5$
Exact Mass: 295.1420
Molecular Weight: 295.3350

5-(Furan-2-ylmethylene)-2,2-dimethyl-1,3-dioxane-4,6-dione (1.00 g, 4.50 mmol, M = 222.20 g/mol) was dissolved in THF (50 mL) and diethylamine (0.33 g, M = 73.14 g/mol) was added. The solution was stirred at room temperature for 10 min. THF was partially evaporated. Addition of ether precipitated a black solid that was filtered off, washed several times with ether and dried under vacuum (1.27 g, 96% yield). ^{1}H-NMR (400 MHz, $CDCl_3$) δ 11.41 (s, 1H), 7.27 (d, *J* = 12.3 Hz, 1H), 7.04 (s, 1H), 6.77–6.68 (m, 1H), 6.05 (t, *J* = 12.4 Hz, 1H), 3.49 (q, *J* = 7.2 Hz, 4H), 1.70 (s, 6H), 1.30 (ddd, *J* = 19.3, 13.3, 6.0 Hz, 6H); ^{13}C-NMR (101 MHz, $CDCl_3$) δ 167.1, 165.3, 156.9, 151.3, 144.9, 139.0, 103.4, 102.2, 90.5, 51.9, 44.1, 26.7, 14.5, 12.3; HRMS (ESI MS) *m*/*z*: theor: 295.1420 found: 295.1418 ($[M]^+$ detected)

Synthesis of 5-((2*Z*,4*E*)-2-hydroxy-5-(piperidin-1-yl)penta-2,4-dien-1-ylidene)-2,2-dimethyl-1,3-dioxane-4,6-dione (**DASA7**)

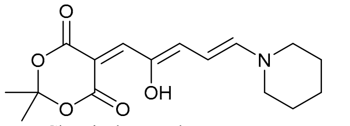

Chemical Formula: $C_{16}H_{21}NO_5$
Exact Mass: 307,1420
Molecular Weight: 307,3460

5-(Furan-2-ylmethylene)-2,2-dimethyl-1,3-dioxane-4,6-dione (0.75 g, 3.375 mmol, M = 222.20 g/mol) was dissolved in THF (40 mL) and piperidine (0.287 g, M = 85.15 g/mol) was added. The solution was stirred at room temperature for 10 min. THF was partially evaporated. Addition of ether precipitated a black solid that was filtered off, washed several times with ether and dried under vacuum (674 mg, 65% yield, blue solid). ^{1}H-NMR (300 MHz, Acetone) δ 11.46 (s, 1H), 7.81 (d, *J* = 12.0 Hz, 1H), 7.01 (dd, *J* = 12.6, 1.3 Hz, 1H), 6.78 (s, 1H), 6.17 (t, *J* = 12.3 Hz, 1H), 3.77 (m, 4H), 1.80 (m, 6H), 1.62 (s, 6H).; HRMS (ESI MS) *m*/*z*: theor: 307.1420 found: 307.1425 ($[M]^+$ detected)

Synthesis of 5-((2*Z*,4*E*)-5-(*bis*(2-hydroxyethyl)amino)-2-hydroxypenta-2,4-dien-1-ylidene)-2,2-dimethyl-1,3-dioxane-4,6-dione (**DASA8**)

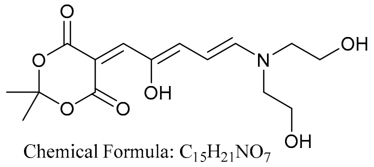

Chemical Formula: $C_{15}H_{21}NO_7$
Exact Mass: 327,1318
Molecular Weight: 327,3330

5-(Furan-2-ylmethylene)-2,2-dimethyl-1,3-dioxane-4,6-dione (0.75 g, 3.375 mmol, M = 222.20 g/mol) was dissolved in THF (40 mL) and diethanolamine (0.355 g, M = 105.14 g/mol) was added. The solution was stirred at room temperature for 10 min. THF was partially evaporated. Addition of ether precipitated a black solid that was filtered off, washed several times with ether and dried under vacuum (861 mg, 78% yield, blue solid). ^{1}H-NMR ($CDCl_3$) δ 11.44 (s, 1H), 7.85 (d, *J* = 12.1 Hz, 1H), 7.02 (d, *J* = 12.6 Hz, 1H), 6.84 (s, 1H), 6.17 (t, *J* = 12.4 Hz, 1H), 4.30 (s, 2H), 3.95–3.86 (m, 4H), 3.86–3.78 (m, 4H), 1.62 (s, 6H); HRMS (ESI MS) *m*/*z*: theor: 327.1318 found: 327.1322 ($[M]^+$ detected); Anal. Calc. for $C_{15}H_{21}NO_7$: C, 55.0; H, 6.5; O, 34.2; Found: C, 55.1; H, 6.5; O, 34.5%.

Synthesis of 5-((2*Z*,4*E*)-5-(dibenzylamino)-2-hydroxypenta-2,4-dien-1-ylidene)-2,2-dimethyl-1,3-dioxane-4,6-dione (**DASA9**)

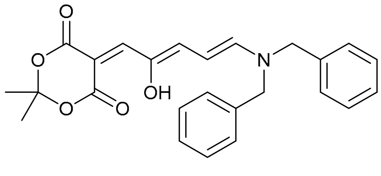

Chemical Formula: $C_{25}H_{25}NO_5$
Exact Mass: 419,1733
Molecular Weight: 419,4770

5-(Furan-2-ylmethylene)-2,2-dimethyl-1,3-dioxane-4,6-dione (0.75 g, 3.375 mmol, M = 222.20 g/mol) was dissolved in THF (40 mL) and dibenzylamine (0.66 g, M = 197.28 g/mol) was added. The solution was stirred at room temperature for 10 min. THF was partially evaporated. Addition of ether precipitated a black solid that was filtered off, washed several times with ether and dried under vacuum (920 mg, 65% yield, blue solid). ^{1}H-NMR ($CDCl_3$) δ 11.28 (s, 1H), 7.49 (d, *J* = 12.4 Hz, 1H), 7.43–7.27 (m, 5H), 7.24–7.16 (m, 6H), 6.74 (d, *J* = 11.9 Hz, 1H), 6.28 (t, *J* = 12.3 Hz, 1H), 4.51 (s, 4H), 1.71 (s, 6H); ^{13}C-NMR ($CDCl_3$) δ 166.8, 164.9, 156.8, 149.7, 145.6, 142.1, 129.3, 128.8, 128.6, 128.5, 128.4, 128.0, 127.5, 103.7, 101.8, 93.0, 26.8; HRMS (ESI MS) *m*/*z*: theor: 419.1733 found: 419.1736 ($[M]^+$ detected)

Synthesis of 5-((2*Z*,4*E*)-2-hydroxy-5-(methyl(phenyl)amino)penta-2,4-dien-1-ylidene)-2,2-dimethyl-1,3-dioxane-4,6-dione (**DASA10**)

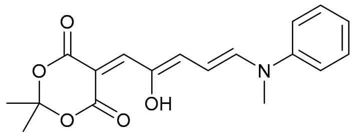

Chemical Formula: $C_{18}H_{19}NO_5$
Exact Mass: 329,1263
Molecular Weight: 329,3520

5-(Furan-2-ylmethylene)-2,2-dimethyl-1,3-dioxane-4,6-dione (0.75 g, 3.375 mmol, M = 222.20 g/mol) was dissolved in THF (40 mL) and N-methylaniline (0.36 g, M = 107.16 g/mol) was added. The solution was stirred at room temperature for 10 min. THF was partially evaporated. Addition of ether precipitated a black solid that was filtered off, washed several times with ether and dried under vacuum (422 mg, 38% yield, blue solid). ^{1}H-NMR ($CDCl_3$) δ: 4.74 (s, 2H), 4.78 (s, 2H), 6.62 (t, 1H, *J* = 12.2 Hz), 7.10 (dd, 1H, *J* = 12.6 Hz, *J* = 1.5 Hz), 7.28-7.44 (m, 12H), 7.46-7.50 (m, 1H), 7.52–7.57 (m, 2H), 8.32 (d, 1H, *J* = 11.9 Hz), 8.38–8.40 (m, 1H), 12.13 (s, 1H); ^{13}C-NMR ($CDCl_3$) δ: 52.9, 61.3, 107.1, 113.7, 117.8, 118.1, 122.4, 124.0, 127.8, 128.7, 129.2, 129.3, 129.7, 129.9, 130.0, 132.5, 134.0, 135.1, 135.3, 137.1, 141.6, 149.6, 153.8, 159.5, 163.6, 191.7; HRMS (ESI MS) m/z: theor: 329.1263 found: 329.1259 ($[M]^{+\cdot}$ detected); Anal. Calc. for $C_{18}H_{19}NO_5$: C, 65.6; H, 5.8; O, 24.3; Found: C, 65.6; H, 5.9; O, 24.5%.

Synthesis of 5-((2*Z*,4*E*)-5-(diethylamino)-2-hydroxypenta-2,4-dien-1-ylidene)-1,3-dimethyl pyrimidine-2,4,6(1*H*,3*H*,5*H*)-trione (**DASA11**)

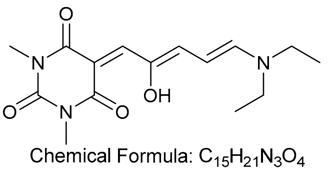

Chemical Formula: $C_{15}H_{21}N_3O_4$
Exact Mass: 307,1532
Molecular Weight: 307,3500

5-(Furan-2-ylmethylene)-1,3-dimethylpyrimidine-2,4,6(1*H*,3*H*,5*H*)-trione (1.0 g, 4.27 mmol, M = 234.21 g/mol) was dissolved in THF (50 mL) and diethylamine (0.312 g, M = 73.14 g/mol) was added. The solution was stirred at room temperature for 10 min. THF was partially evaporated. Addition of ether precipitated a black solid that was filtered off, washed several times with ether and dried under vacuum (1.05 g, 80% yield, blue solid). ^{1}H-NMR ($CDCl_3$) δ 12.48 (s, 1H), 7.23 (d, *J* = 12.3 Hz, 1H), 7.10 (s, 1H), 6.73 (d, *J* = 12.1 Hz, 1H), 6.05 (t, *J* = 12.3 Hz, 1H), 3.53–3.41 (m, 4H), 3.32 (s, 3H), 3.31 (s, 3H), 1.39–1.21 (m, 6H); HRMS (ESI MS) *m*/*z*: theor: 307.1532 found: 307.1536 ($[M]^+$ detected); Anal. Calc. for $C_{15}H_{21}N_3O_4$: C, 58.6; H, 6.9; O, 20.8; Found: C, 58.6; H, 6.9; O, 20.5%.

Synthesis of 5-((2*Z*,4*E*)-5-(*bis*(2-hydroxyethyl)amino)-2-hydroxypenta-2,4-dien-1-ylidene)-1,3-dimethylpyrimidine-2,4,6(1*H*,3*H*,5*H*)-trione (**DASA12**)

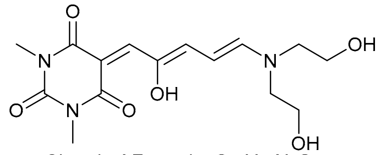

Chemical Formula: $C_{15}H_{21}N_3O_6$
Exact Mass: 339,1430
Molecular Weight: 339,3480

5-(Furan-2-ylmethylene)-1,3-dimethylpyrimidine-2,4,6(1*H*,3*H*,5*H*)-trione (0.75 g, 3.20 mmol, M = 234.21 g/mol) was dissolved in THF (50 mL) and diethanolamine (0.336 g, M = 105.14 g/mol) was added. The solution was stirred at room temperature for 10 min. THF was partially evaporated. Addition of ether precipitated a black solid that was filtered off, washed several times with ether and dried under vacuum (738 mg, 68% yield, blue solid). ^{1}H-NMR (DMSO-d_6) δ: 12.48 (s, 1H), 8.00 (d, *J* = 11.7 Hz, 1H), 7.18 (d, *J* = 12.0 Hz, 1H), 6.70 (s, 1H), 6.20 (t, *J* = 12.2 Hz, 1H), 5.05 (s, 2H), 3.71–3.64 (m, 8H), 3.30 (s, 6H); ^{13}C-NMR (DMSO-d_6) δ: 164.1, 153.9, 151.2, 144.8, 130.4, 105.8, 93.6, 59.9, 58.1, 58.0, 52.0, 27.8; HRMS (ESI MS) *m*/*z*: theor: 339.1430 found: 339.1434 ($[M]^+$ detected)

Synthesis of 2-((2*Z*,4*E*)-5-(diethylamino)-2-hydroxypenta-2,4-dien-1-ylidene)-1*H*-indene-1,3(2*H*)-dione (**DASA13**)

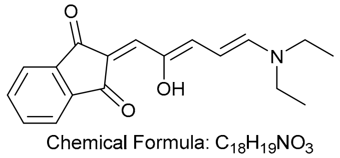

Chemical Formula: $C_{18}H_{19}NO_3$
Exact Mass: 297,1365
Molecular Weight: 297,3540

2-(Furan-2-ylmethylene)-1*H*-indene-1,3(2*H*)-dione (0.75 g, 3.34 mmol, M = 224.22 g/mol) was dissolved in THF (40 mL) and diethylamine (0.244 g, M = 73.14 g/mol) was added. The solution was stirred at room temperature for 10 min. THF was partially evaporated. Addition of ether precipitated a black solid that was filtered off, washed several times with ether and dried under vacuum (884 mg, 89% yield, blue solid). ^{1}H-NMR (deuterated acetone) δ 11.67 (s, *J* = 0.6 Hz, 1H), 7.77 (d, *J* = 12.1 Hz, 1H), 7.61–7.51 (m, 4H), 6.98 (dd, *J* = 12.5, 1.4 Hz, 1H), 6.51 (s, 1H), 6.13 (t, *J* = 12.3 Hz, 1H), 3.69 (q, *J* = 7.2 Hz, 4H), 1.40–1.30 (m, 6H) ; HRMS (ESI MS) *m*/*z*: theor: 297.1365 found: 297.1366 ($[M]^+$ detected); Anal. Calc. for $C_{18}H_{19}NO_3$: C, 72.7; H, 6.4; O, 16.1; Found: C, 72.8; H, 6.6; O, 16.3%.

2. X-Ray Crystallography

Table S1. Crystal data and structure refinement for compounds PP26 (CCDC 1998738).

	**PP26**
Formula	$C_{27}H_{17}N_5O$
Formula weight	427.46
Temperature/K	296.15
Crystal color	Black
Crystal size/mm	0.23 × 0.15 × 0.12
Crystal system	Monoclinic
Space group	$P2_1/n$
a/Å	10.446(3)
b/Å	18.777(4)
c/Å	11.148(3)
α/°	90
β/°	103.141(5)
γ°	90
Volume/Å^3	2129.5(9)
Z, $\rho_{calculated}$/g.cm^{-3}	4, 1.333
μ/mm^{-1}	0.054
Θ range/°	1.71–16.297
Limiting indices	$-10 \le h \le 10$<br>$-18 \le k \le 18$<br>$-10 \le l \le 11$
Collected reflections	7934
Unique reflections	2234<br>[R(int) = 0.1014]
Parameters	299
Goodness-of-fit on F^2	0.991
Final R indices [I>2σ(I)]	R1 = 0.0508<br>wR2 = 0.1062
R indices (all data)	R1 = 0.0990<br>wR2 = 0.1323
Largest diff. peak and hole/e.Å^{-3}	0.16 and −0.24

Data collection and structure refinements:

X-ray analyses of compound **PP26** were performed at 296.15K. X-ray intensity data were collected on a Bruker X8-APEX2 CCD area-detector diffractometer using Mo-K_α radiation (λ = 0.71073 Å) with an optical fiber as collimator. Several sets of narrow data frames (20 sec per frame) were collected at different values of θ for two initial values of ϕ and ω, respectively, using 0.3° increments of ϕ or ω. Data reduction was accomplished using SAINT V8.34a. [1] The substantial redundancy in data allowed a semi-empirical absorption correction (SADABS V2014/5) [2] to be applied, on the basis of multiple measurements of equivalent reflections. The structure was solved by direct methods, developed by successive difference Fourier syntheses, and refined by full-matrix least-squares on all F^2 data using SHELX program suite [3] on the OLEX2 graphical tool.[4] Hydrogen atoms were included in calculated positions and allowed to ride on their parent atoms. The final refinements include anisotropic thermal parameters of all non-hydrogen atoms. The crystal data are given in Table S1. Supporting information is available in CIF format.

PP26 Structure Images:

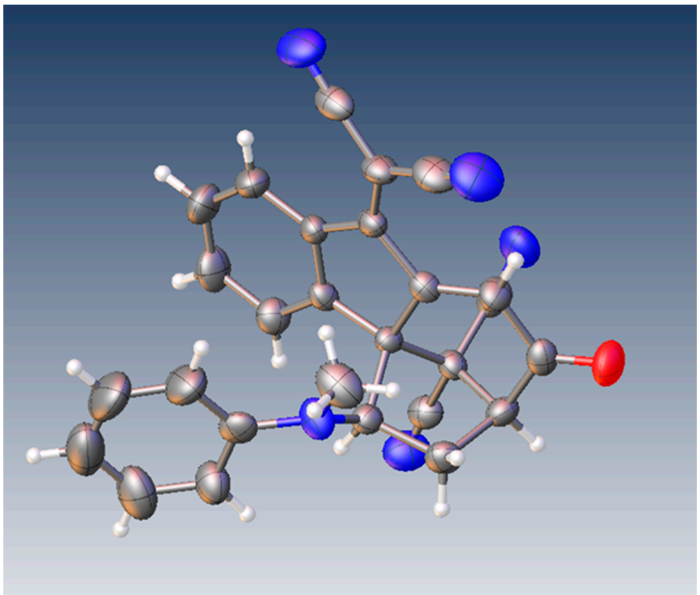

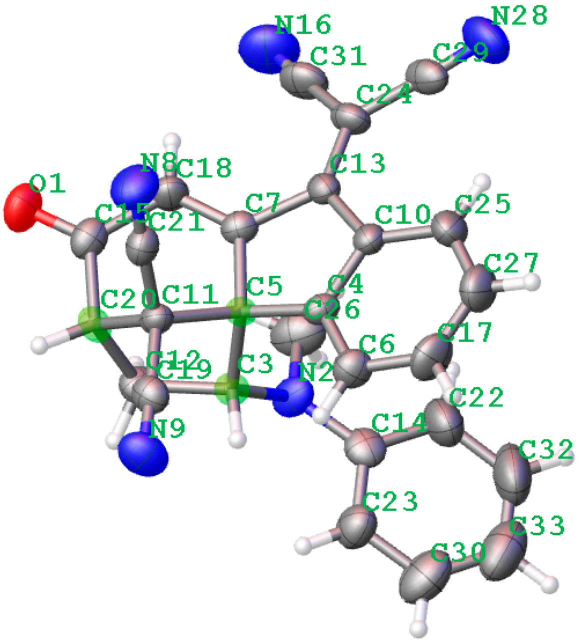

Figure S1. Configuration of the asymmetric carbons: C3 (R), C5 (R), C20 (S).

References

1. SAINT Plus Version 7.53a; Brucker Analytical X-ray Systems: Madison, WI, USA, 2008.
2. Sheldrick, G. M., SADABS, Brucker-Siemens Area detector Absorption and Other Correction, Version 2008/1; Brucker: Madison, WI, USA, 2008.
3. Sheldrick, G. M., A Short History of SHELX. *Acta Crystallogr. A* **2008,** *64*, 112–122.
4. Dolomanov, O. V.; Bourhis, L. J.; Gildea, R. J.; Howard, J. A. K.; Puschmann, H. OLEX2: A Complete Structure Solution, Refinement and Analysis Program. *J. Appl. Crystallogr.* **2009**, *42*, 339–341.
